# Supplementary material for: Tissue-specific responses to TFAM and mtDNA copy number manipulation in prematurely ageing mice
Source: eLife. 2025 Jun 30;14:RP104461. doi: 10.7554/eLife.104461 (PMC12208663; doi:10.7554/eLife.104461)
Supplement: Figure 2—figure supplement 1—source data 4. [file elife-104461-fig2-figsupp1-data4.pdf]

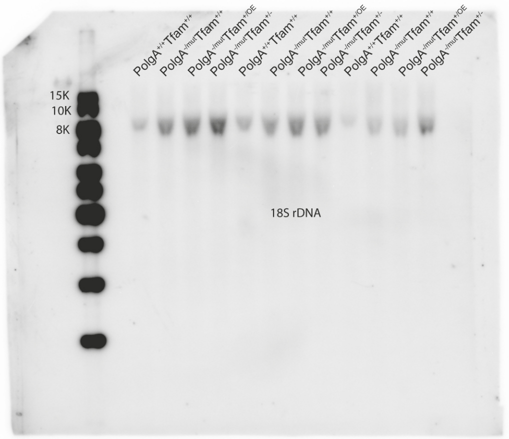

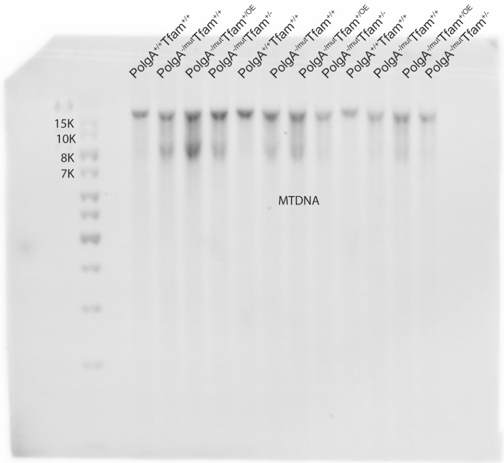

Figure 2 - Figure supplement 1 - Source data 4

Southern blot analysis for Figure 2 - Figure supplement 1B, indicating relevant bands in kilo basepairs (K)
