# Supplementary figures and images for: Tissue-specific responses to TFAM and mtDNA copy number manipulation in prematurely ageing mice
Source: eLife. 2025 Jun 30;14:RP104461. doi: 10.7554/eLife.104461 (PMC12208663; doi:10.7554/eLife.104461)

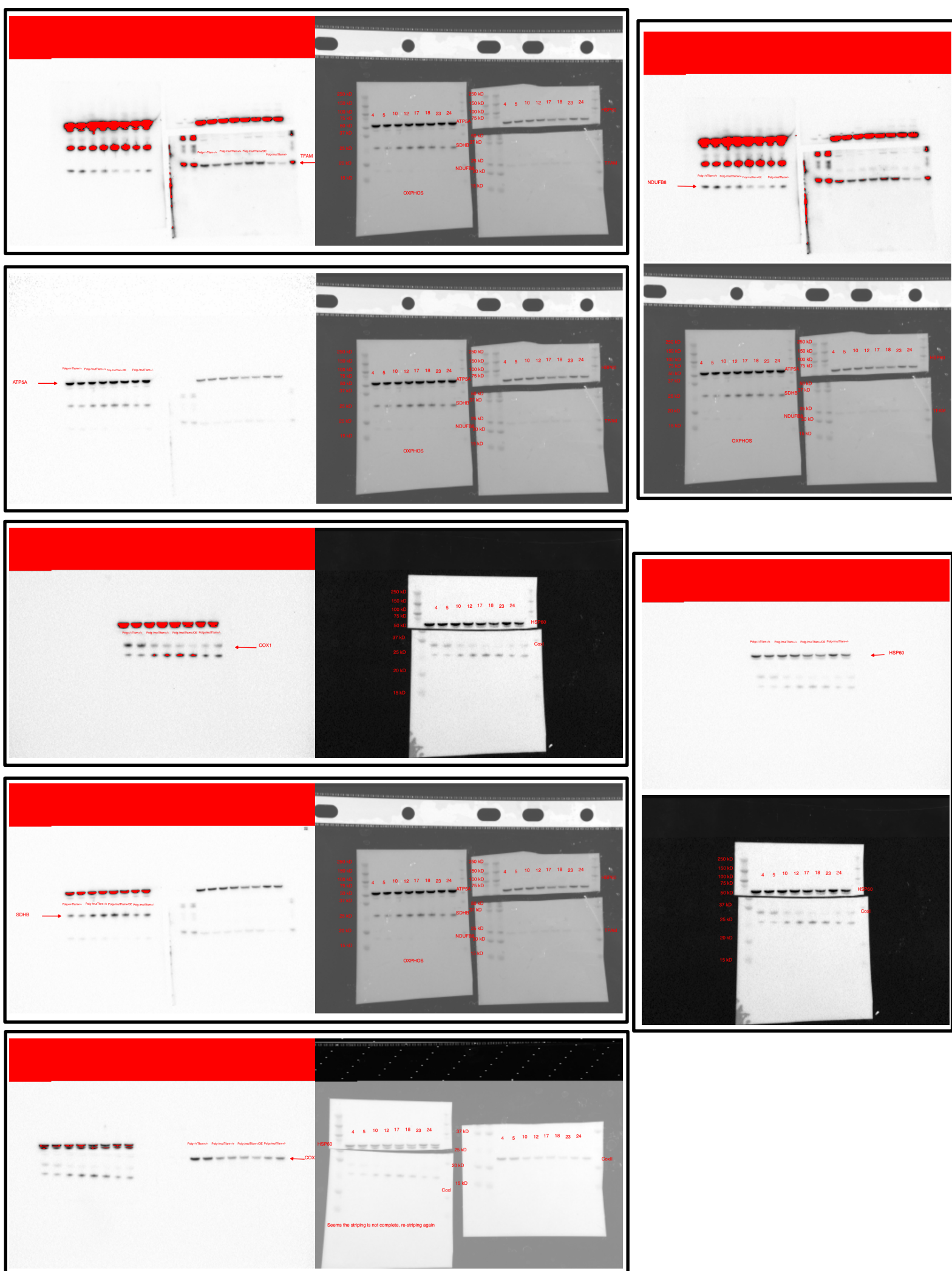

Supplement: Figure 3—source data 3. [file elife-104461-fig3-data3.pdf]

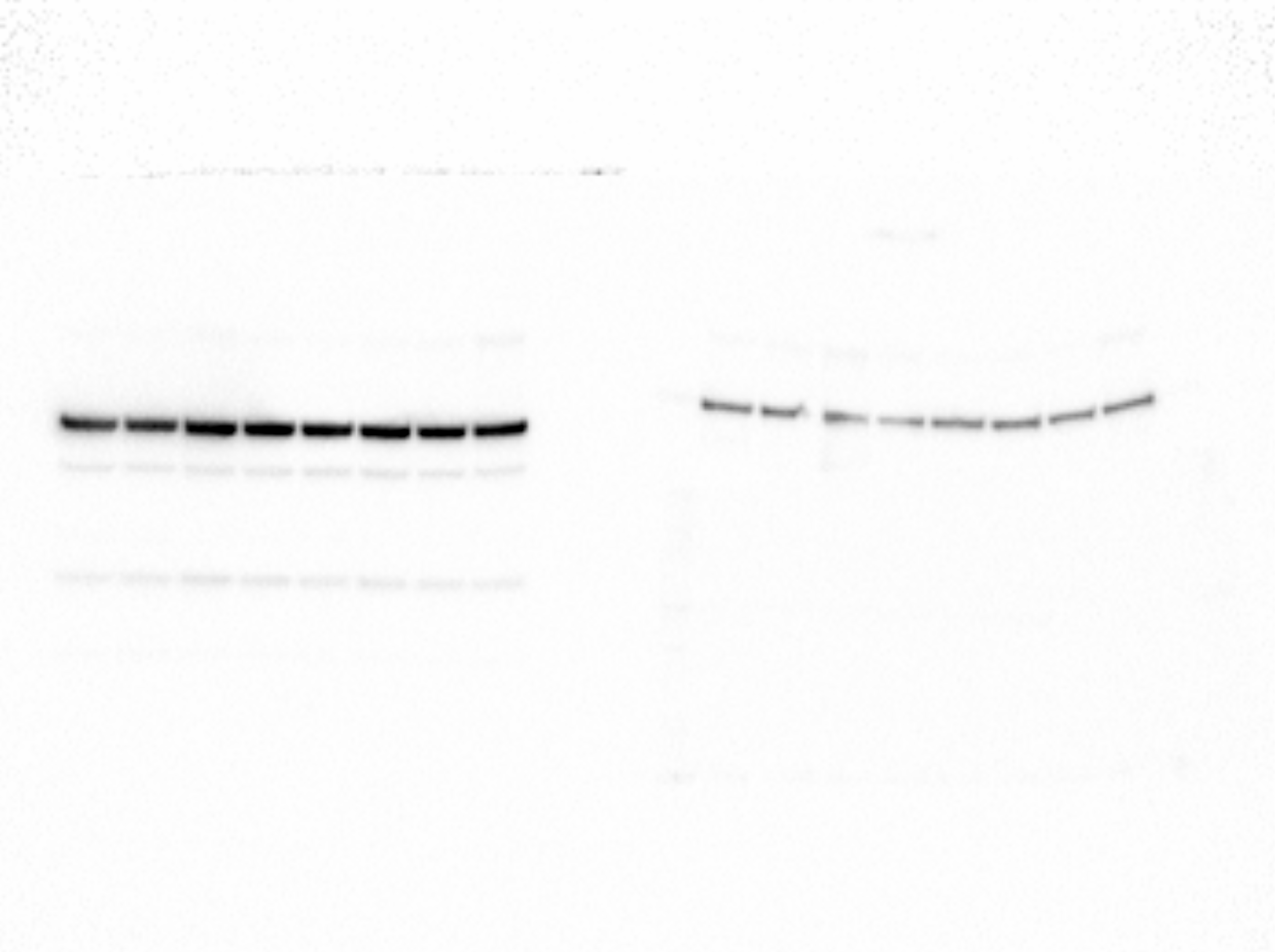

Supplement: Figure 4—source data 2. [file elife-104461-fig4-data2.zip › ATP5A.tif]

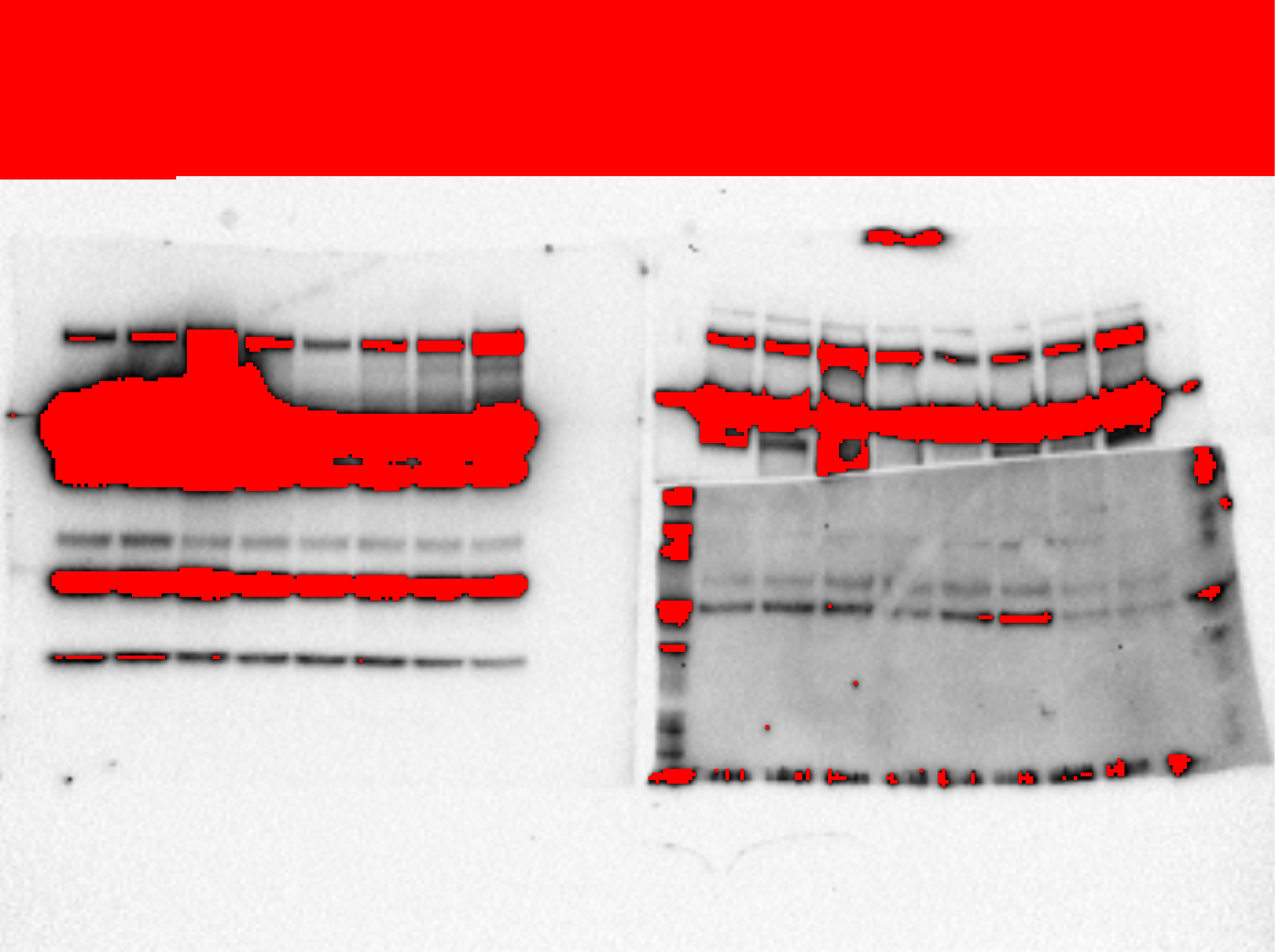

Supplement: Figure 4—source data 2. [file elife-104461-fig4-data2.zip › Cox1.tif]

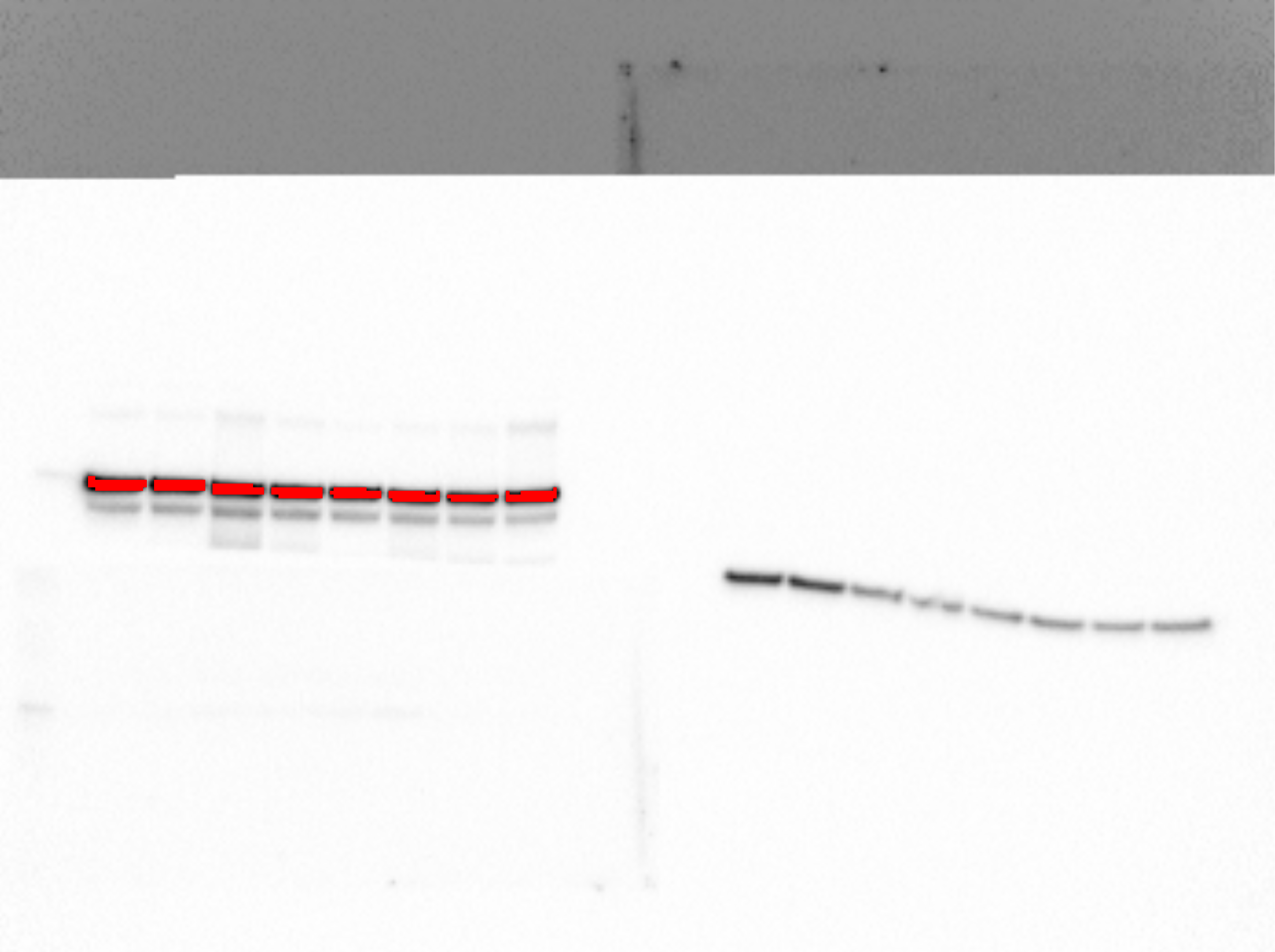

Supplement: Figure 4—source data 2. [file elife-104461-fig4-data2.zip › Cox2.tif]

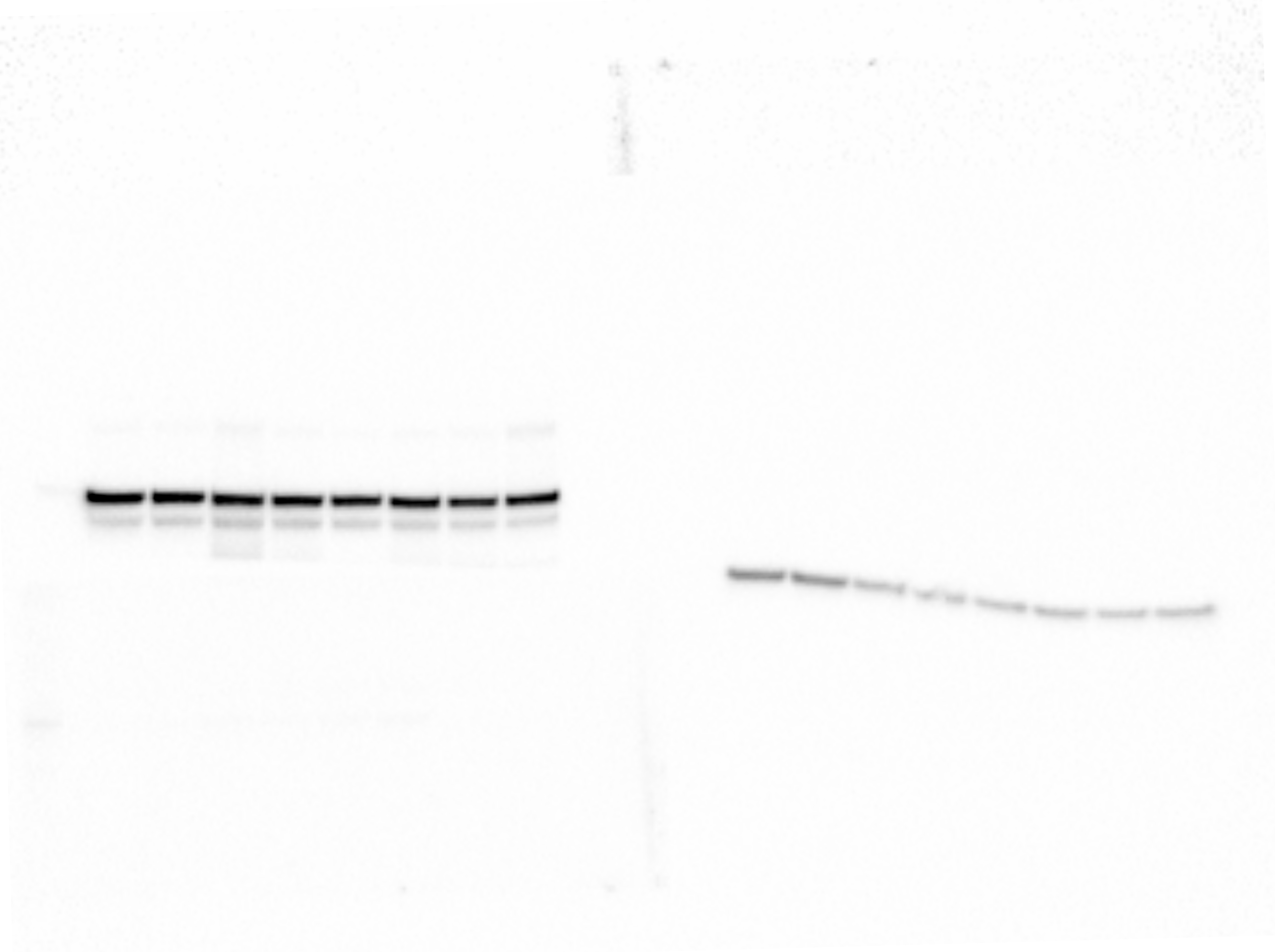

Supplement: Figure 4—source data 2. [file elife-104461-fig4-data2.zip › HSP60.tif]

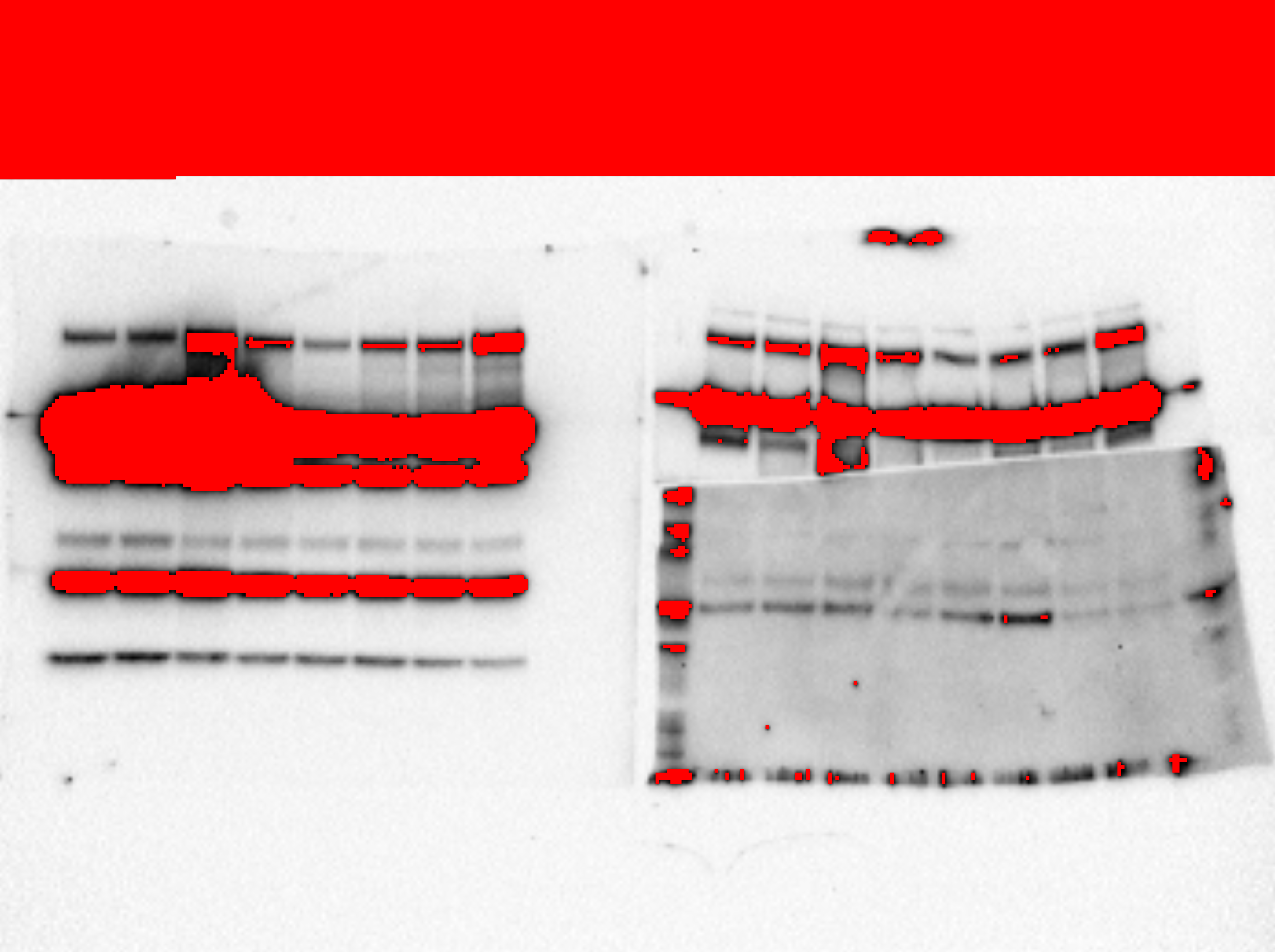

Supplement: Figure 4—source data 2. [file elife-104461-fig4-data2.zip › NDUFB8.tif]

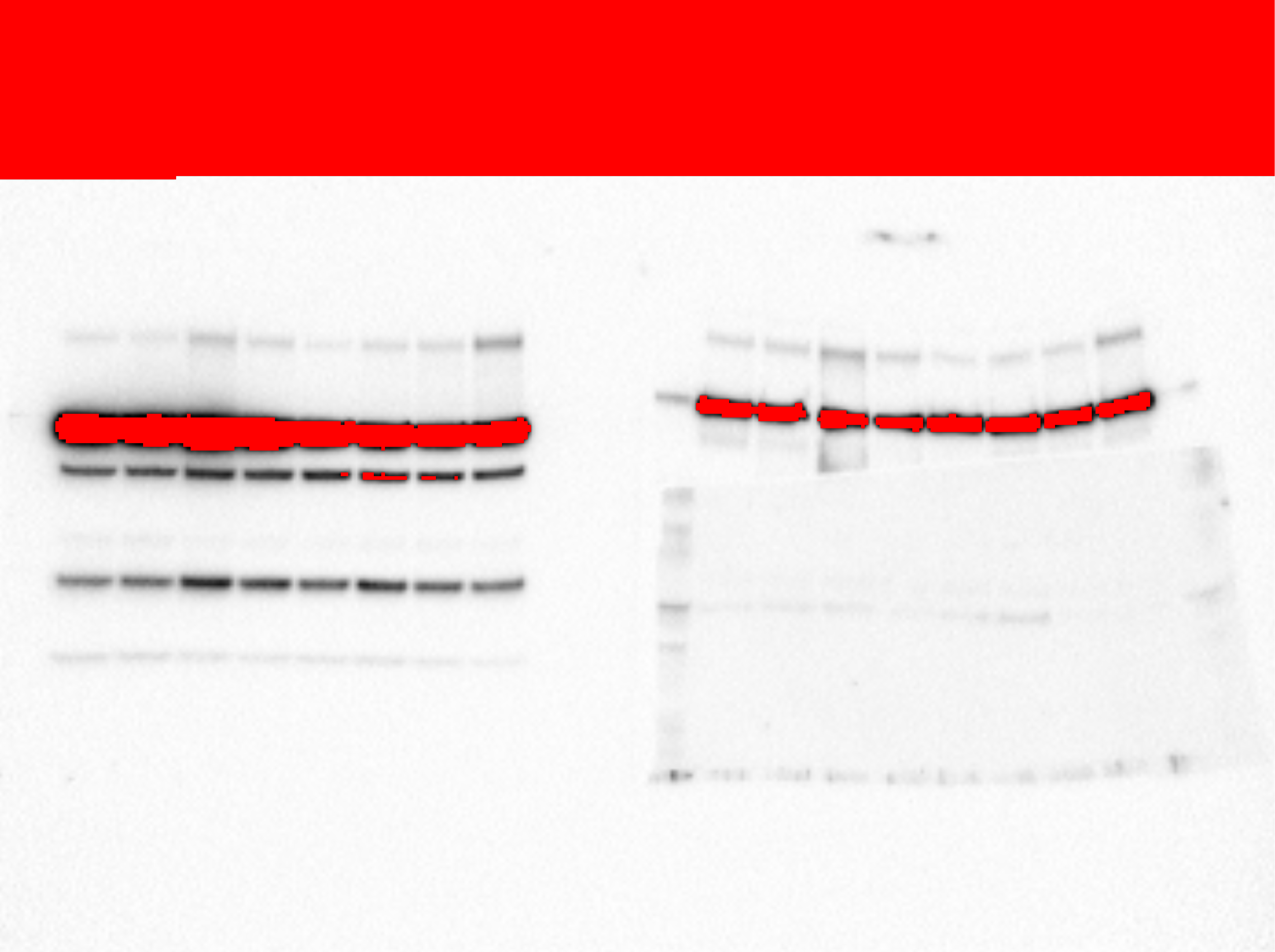

Supplement: Figure 4—source data 2. [file elife-104461-fig4-data2.zip › SDHB.tif]

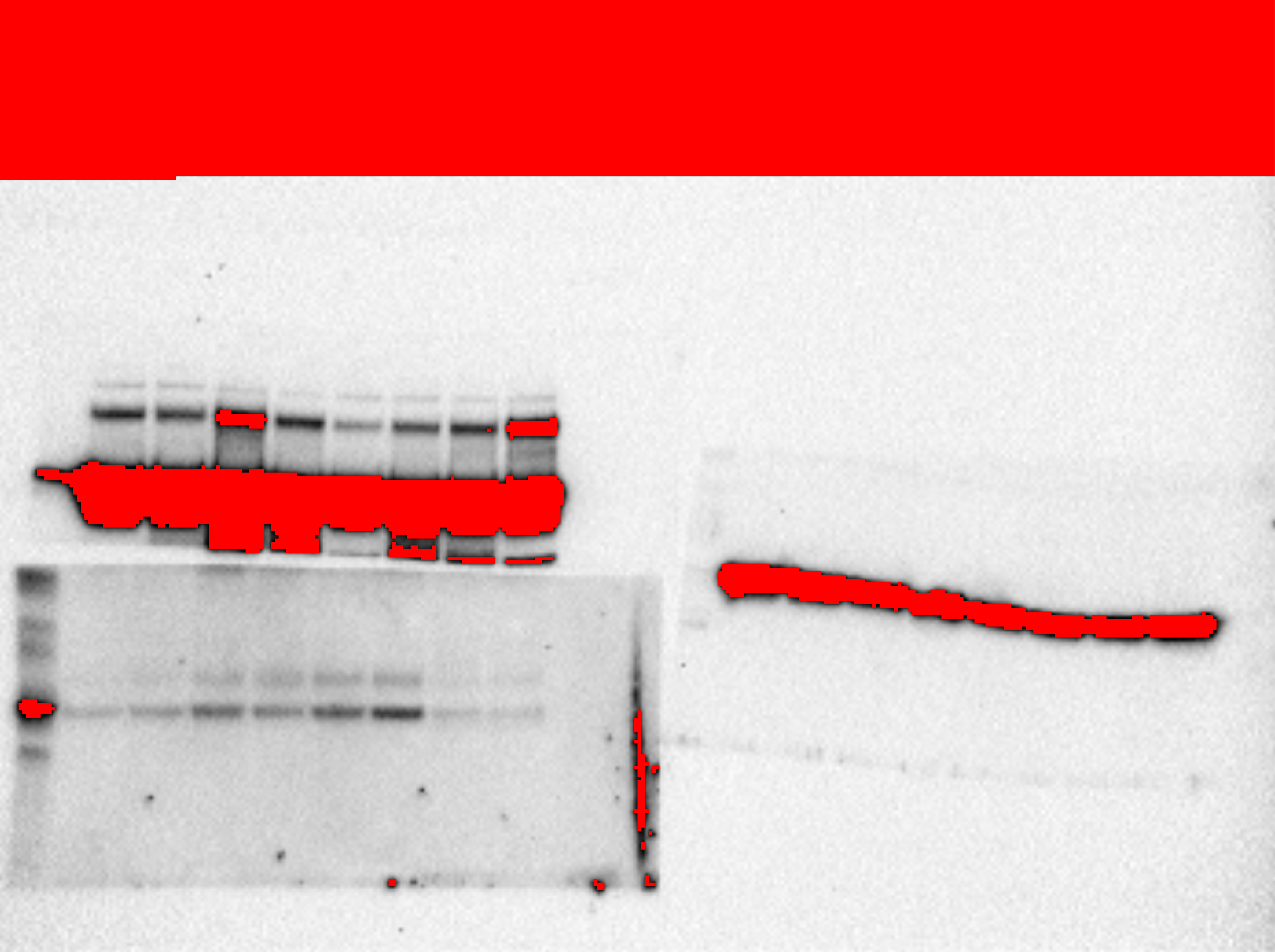

Supplement: Figure 4—source data 2. [file elife-104461-fig4-data2.zip › Tfam.tif]

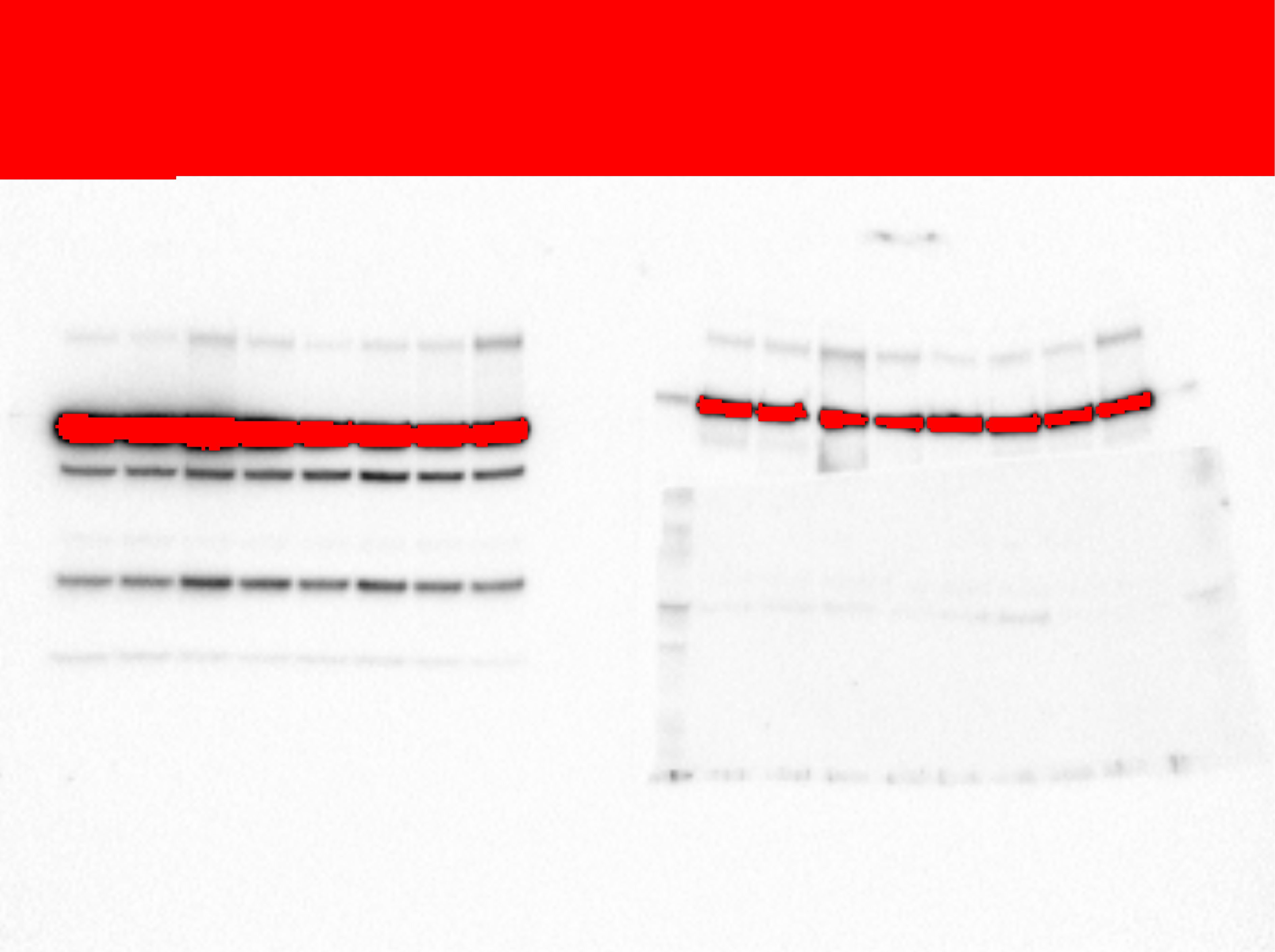

Supplement: Figure 4—source data 2. [file elife-104461-fig4-data2.zip › UQCRC2.tif]

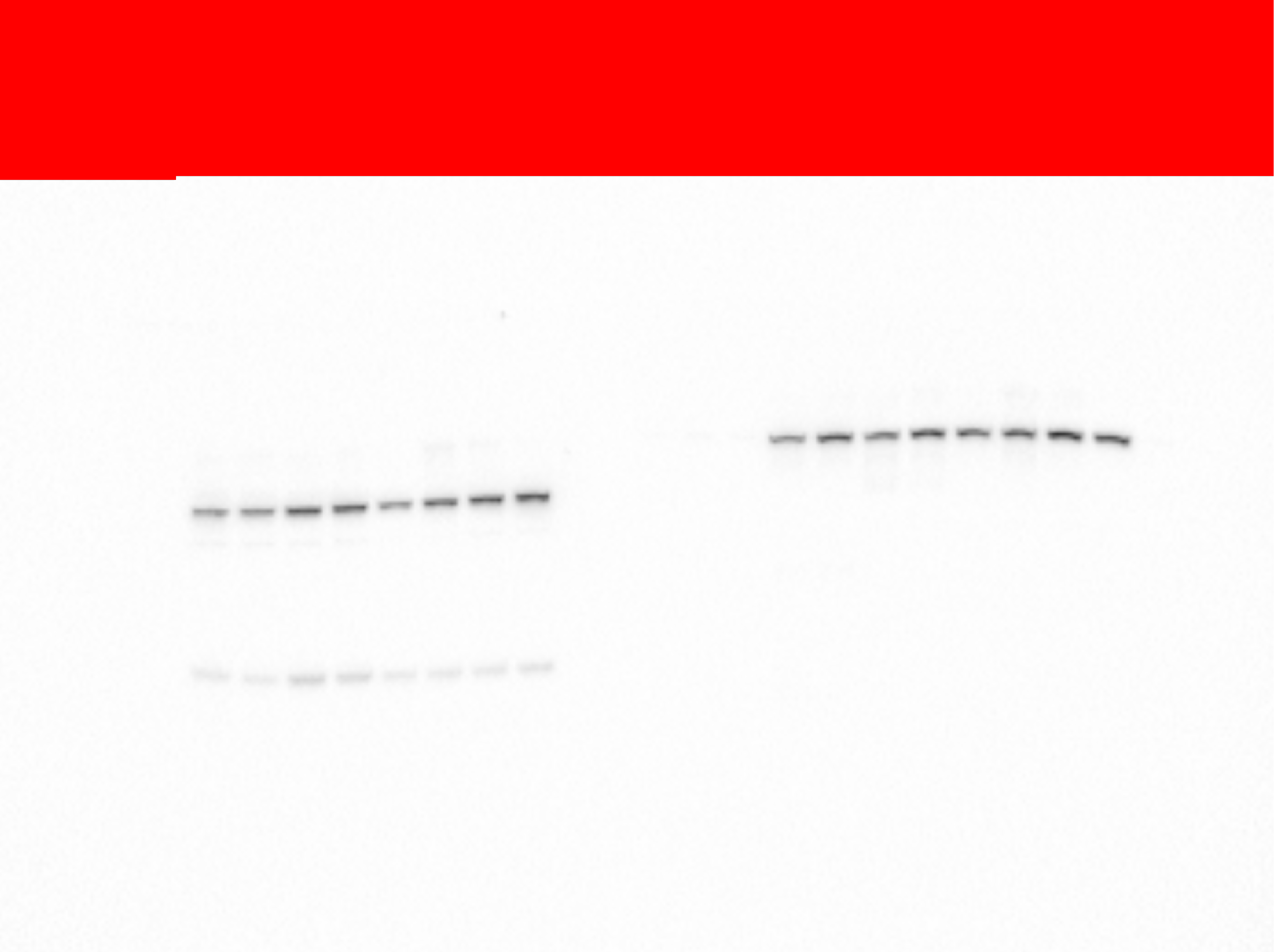

Supplement: Figure 5—source data 2. [file elife-104461-fig5-data2.zip › ATP5A.tif]

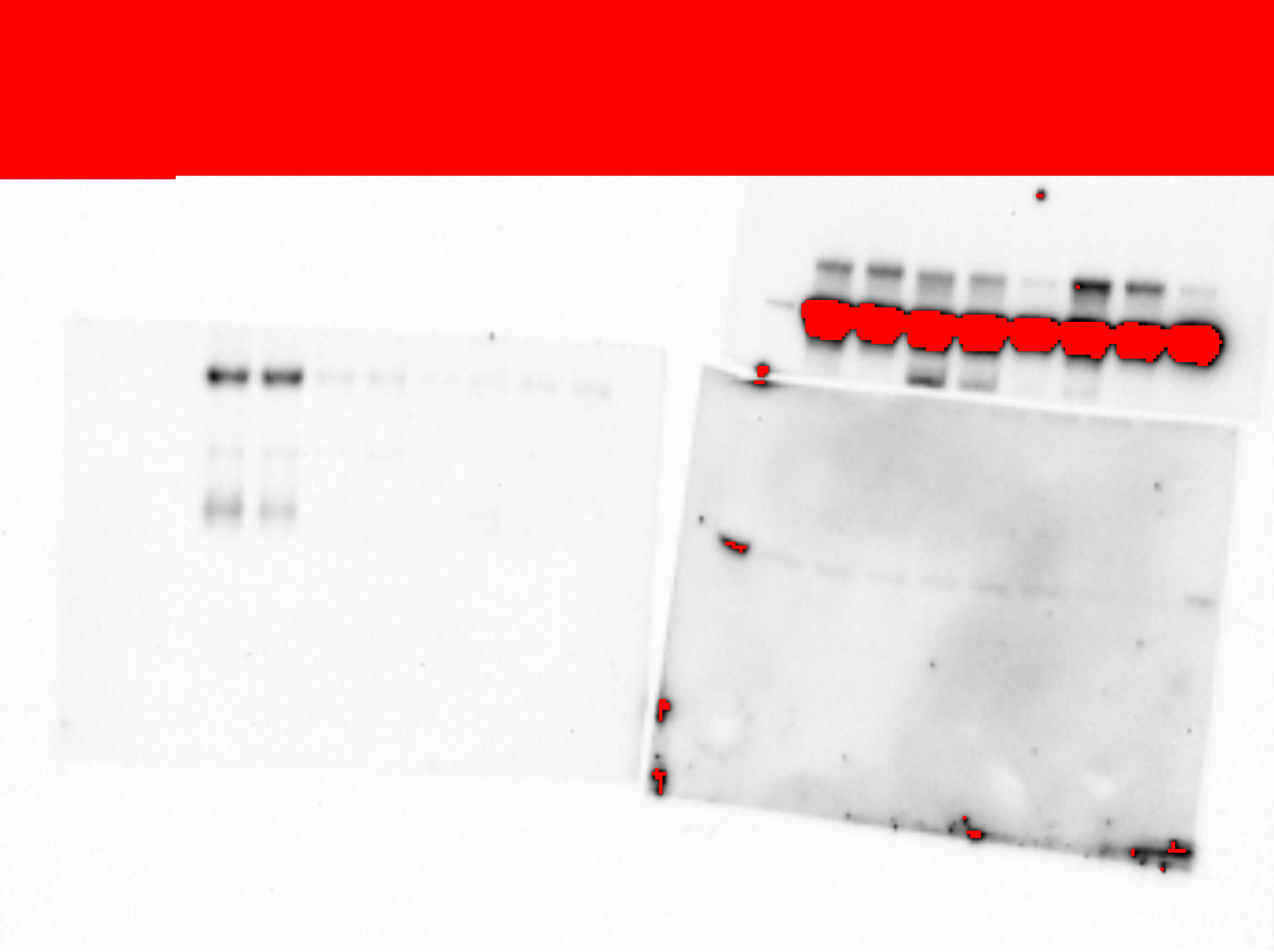

Supplement: Figure 5—source data 2. [file elife-104461-fig5-data2.zip › Cox1.tif]

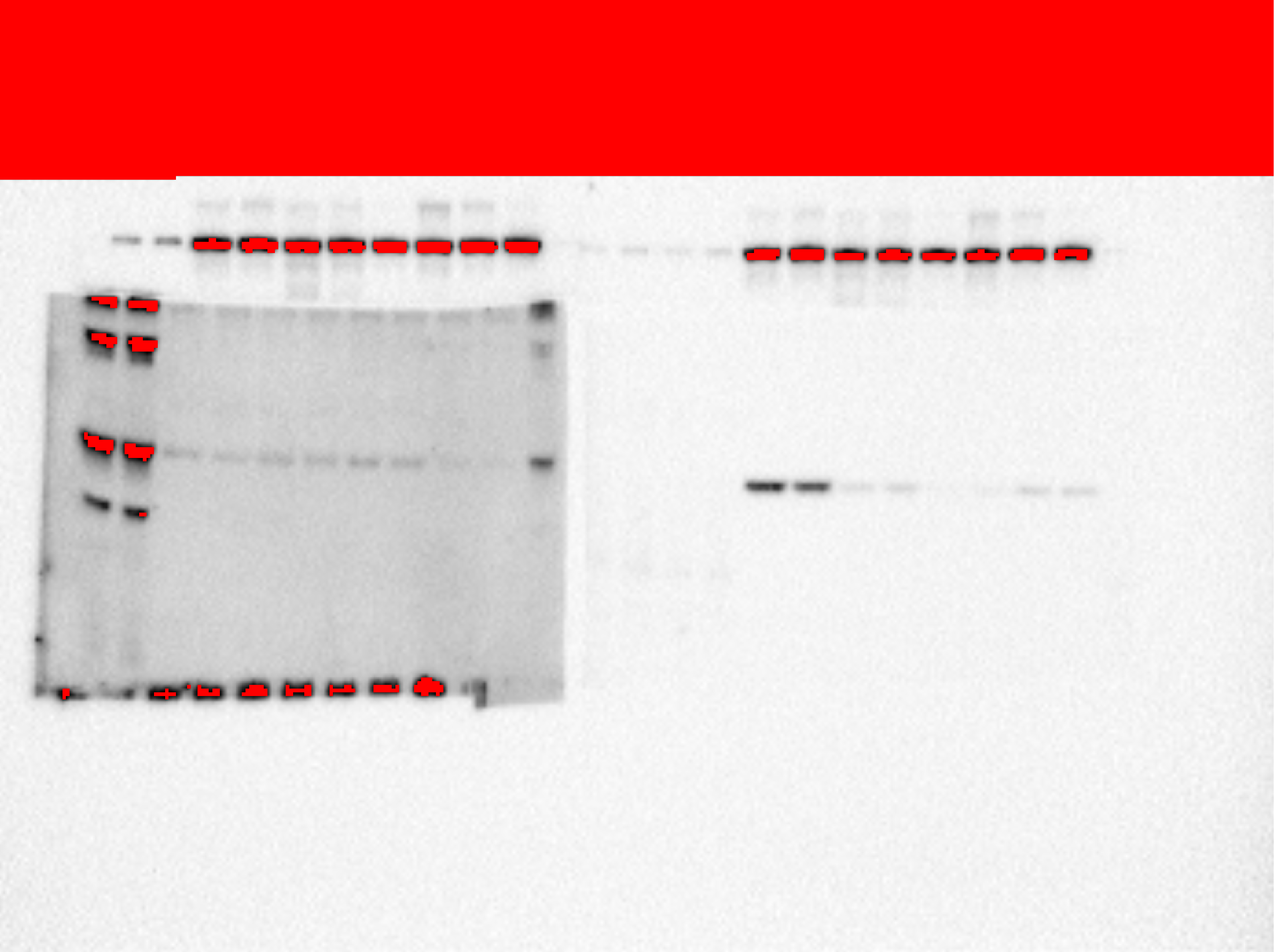

Supplement: Figure 5—source data 2. [file elife-104461-fig5-data2.zip › Cox2.tif]

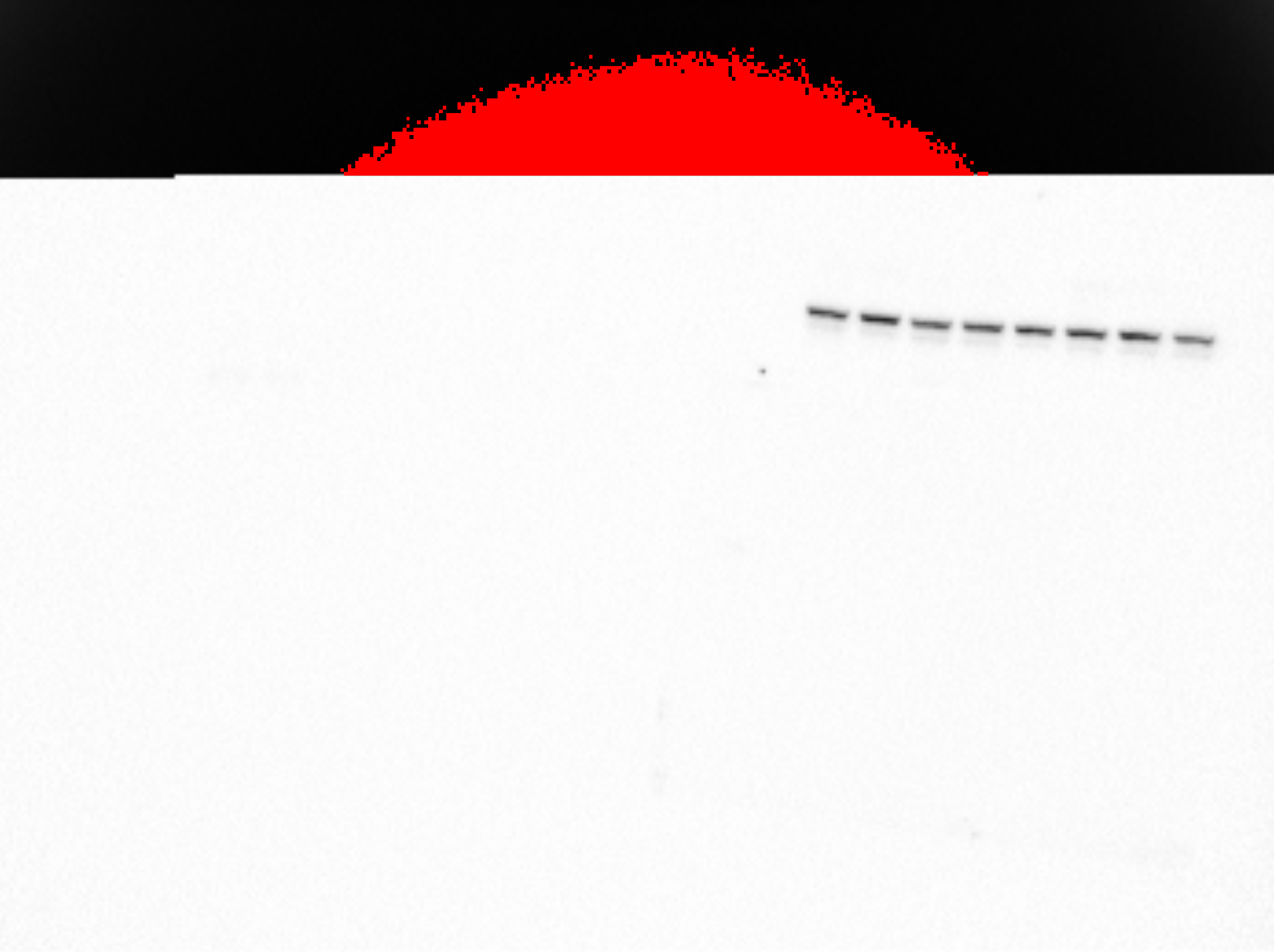

Supplement: Figure 5—source data 2. [file elife-104461-fig5-data2.zip › HSP60.tif]

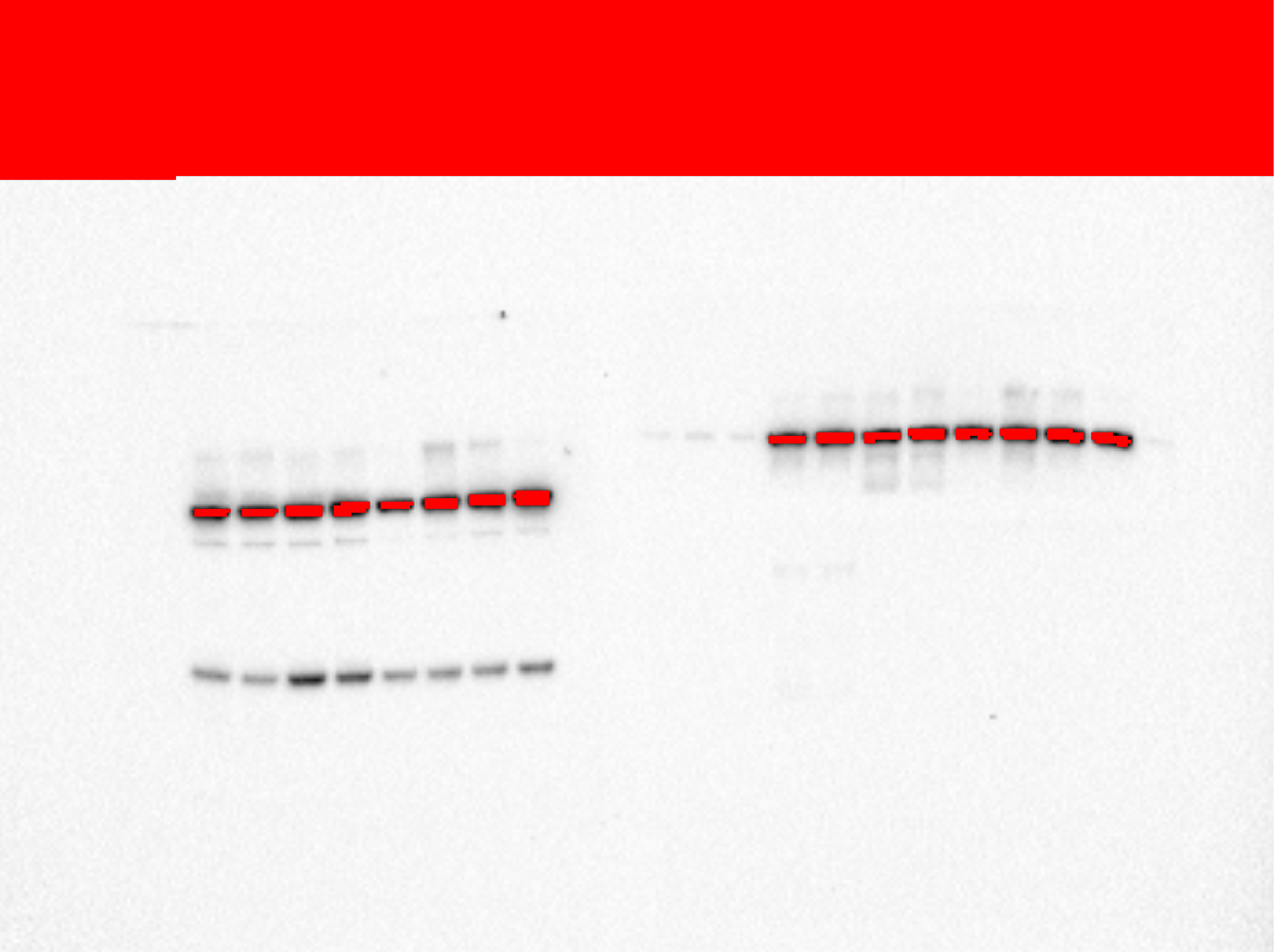

Supplement: Figure 5—source data 2. [file elife-104461-fig5-data2.zip › SDHB.tif]

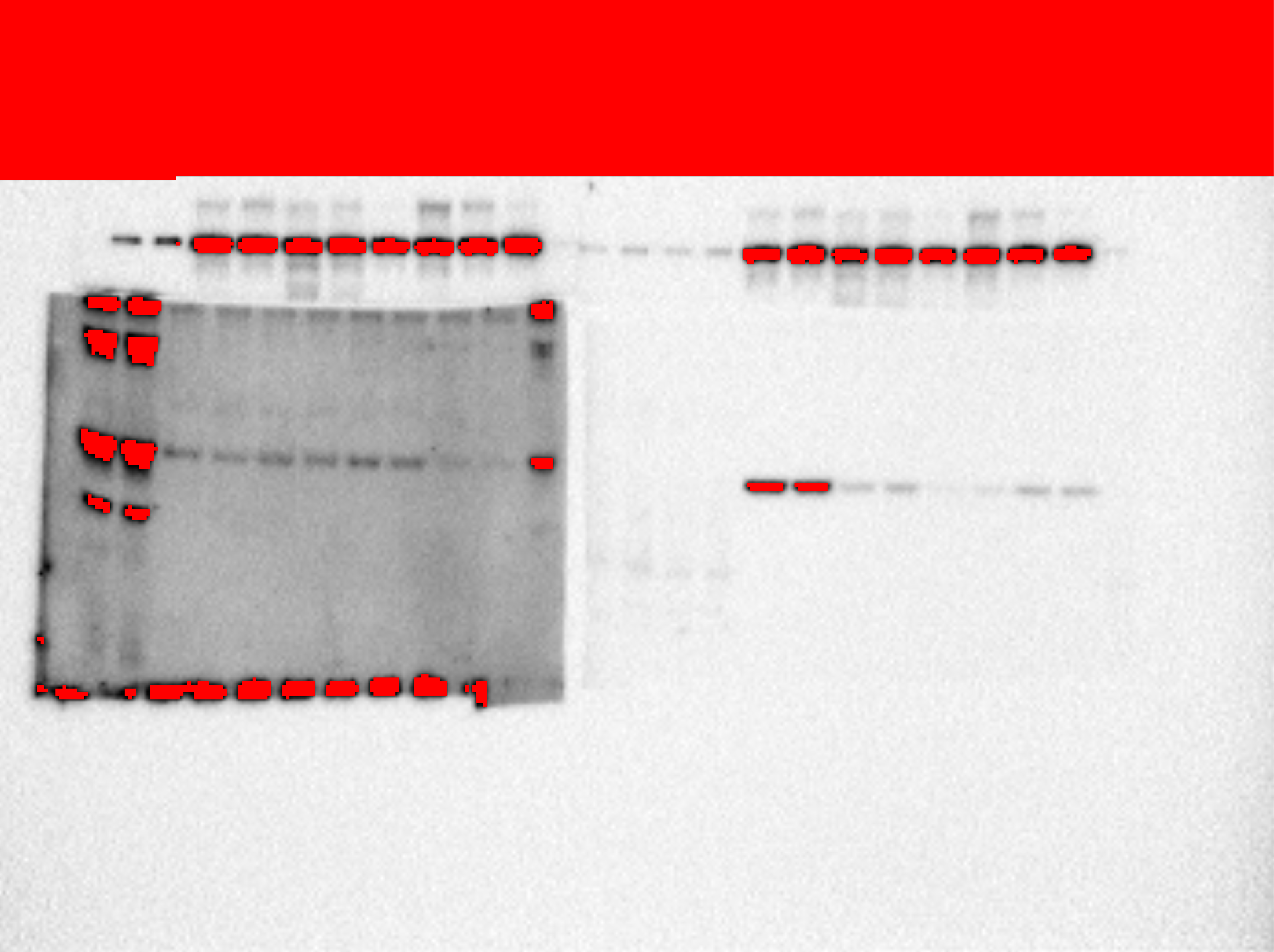

Supplement: Figure 5—source data 2. [file elife-104461-fig5-data2.zip › tfam.tif]

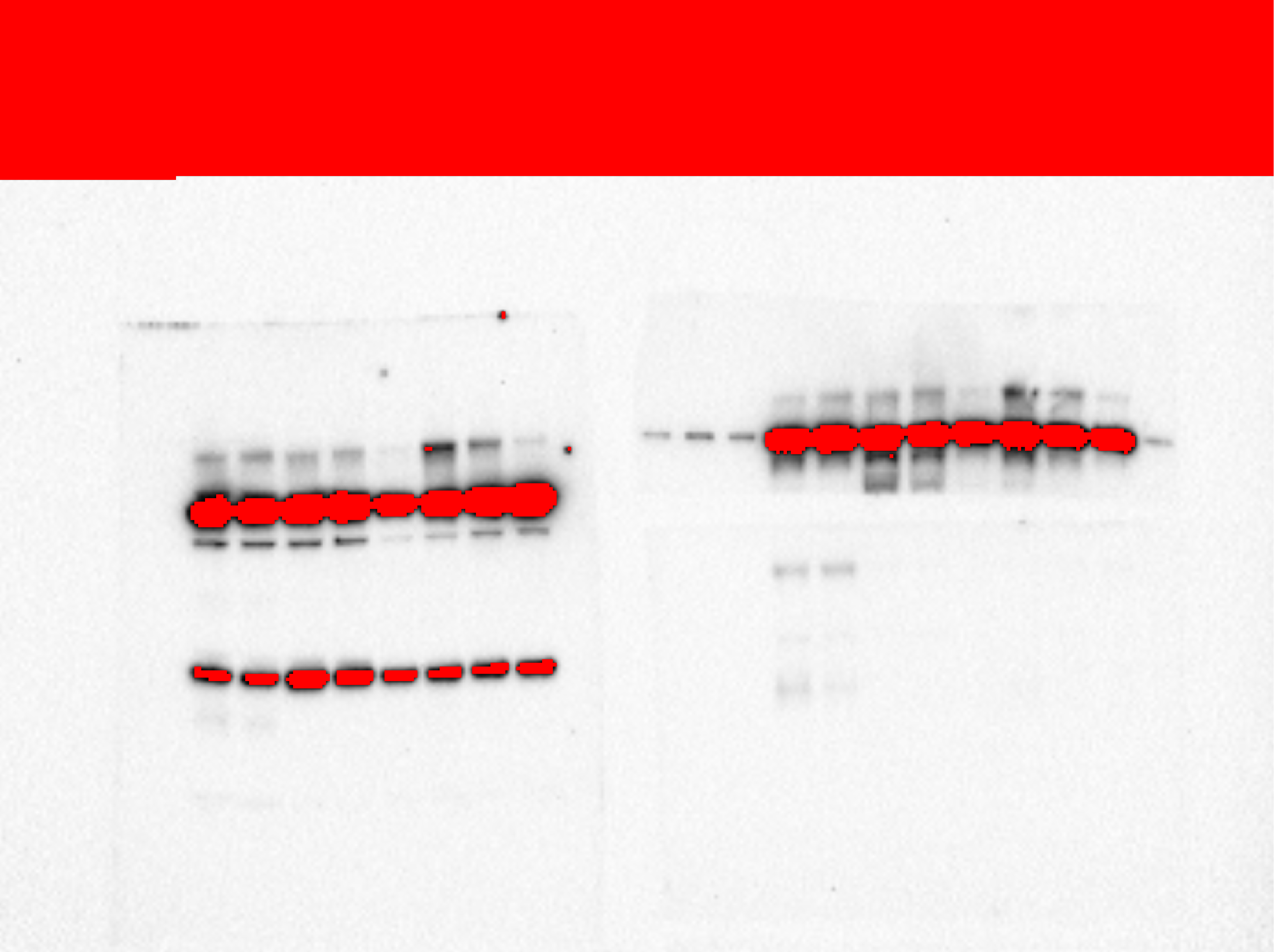

Supplement: Figure 5—source data 2. [file elife-104461-fig5-data2.zip › UQCRC2.tif]

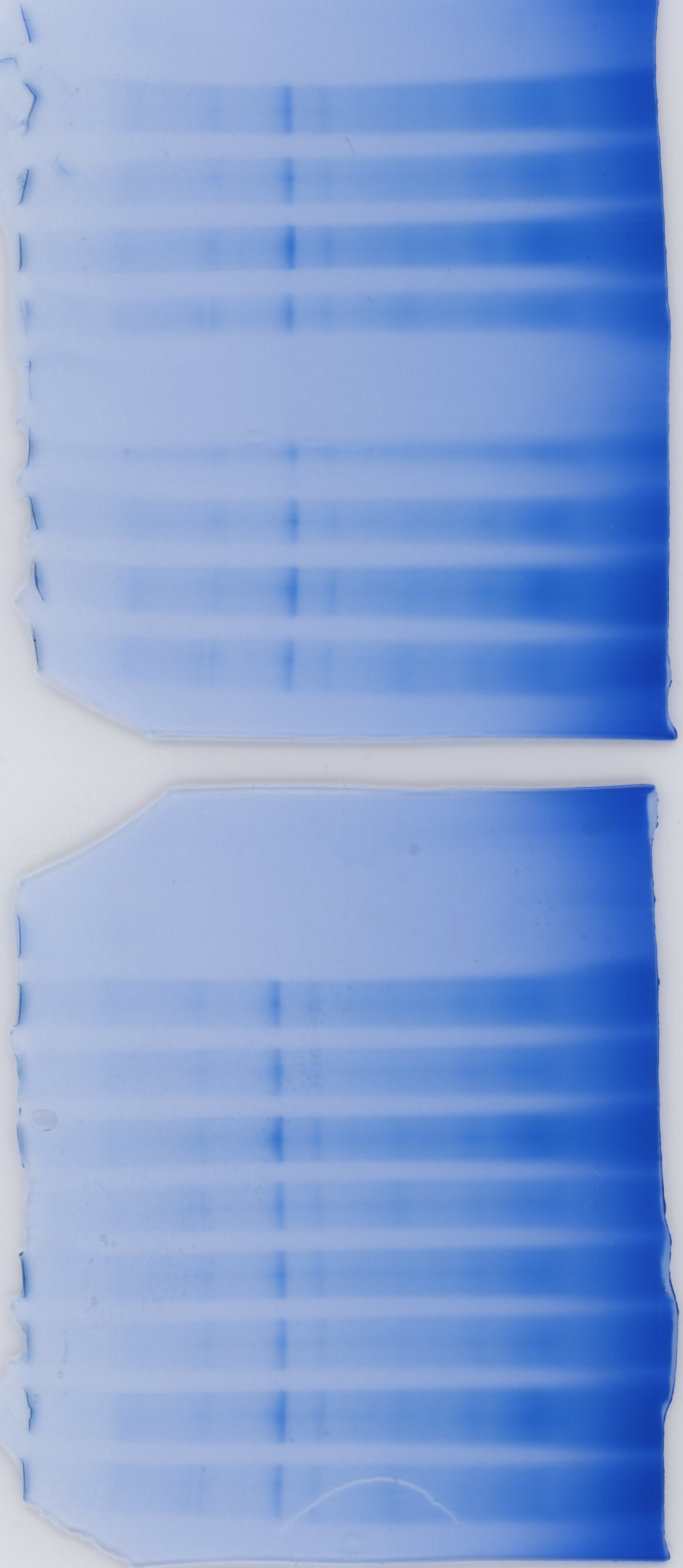

Supplement: Figure 5—source data 3. [file elife-104461-fig5-data3.zip › Figure 5-source data 3/COMMASSIE.tiff]

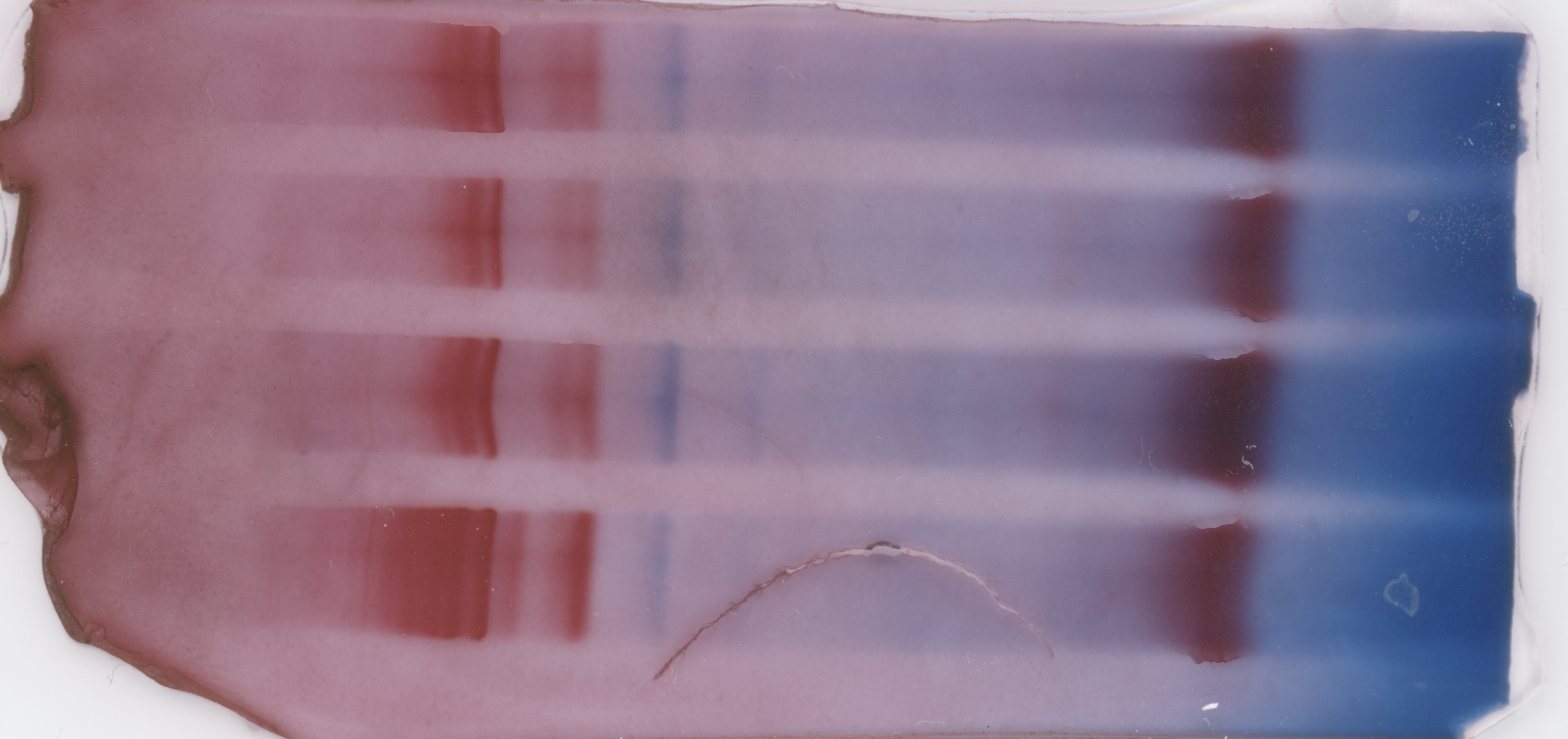

Supplement: Figure 5—source data 3. [file elife-104461-fig5-data3.zip › Figure 5-source data 3/COMPLEX I.tiff]

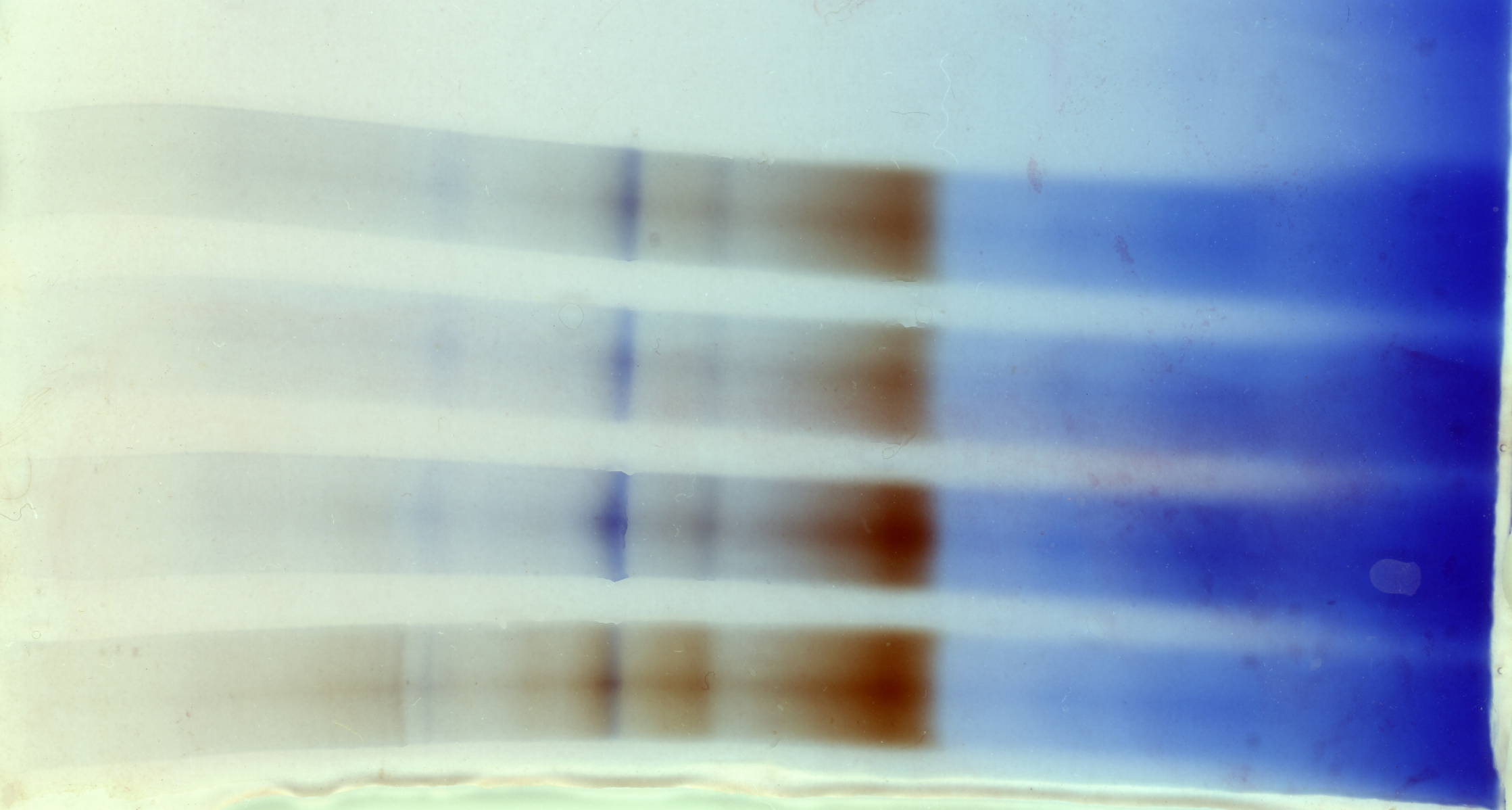

Supplement: Figure 5—source data 3. [file elife-104461-fig5-data3.zip › Figure 5-source data 3/COMPLEX IV.tiff]

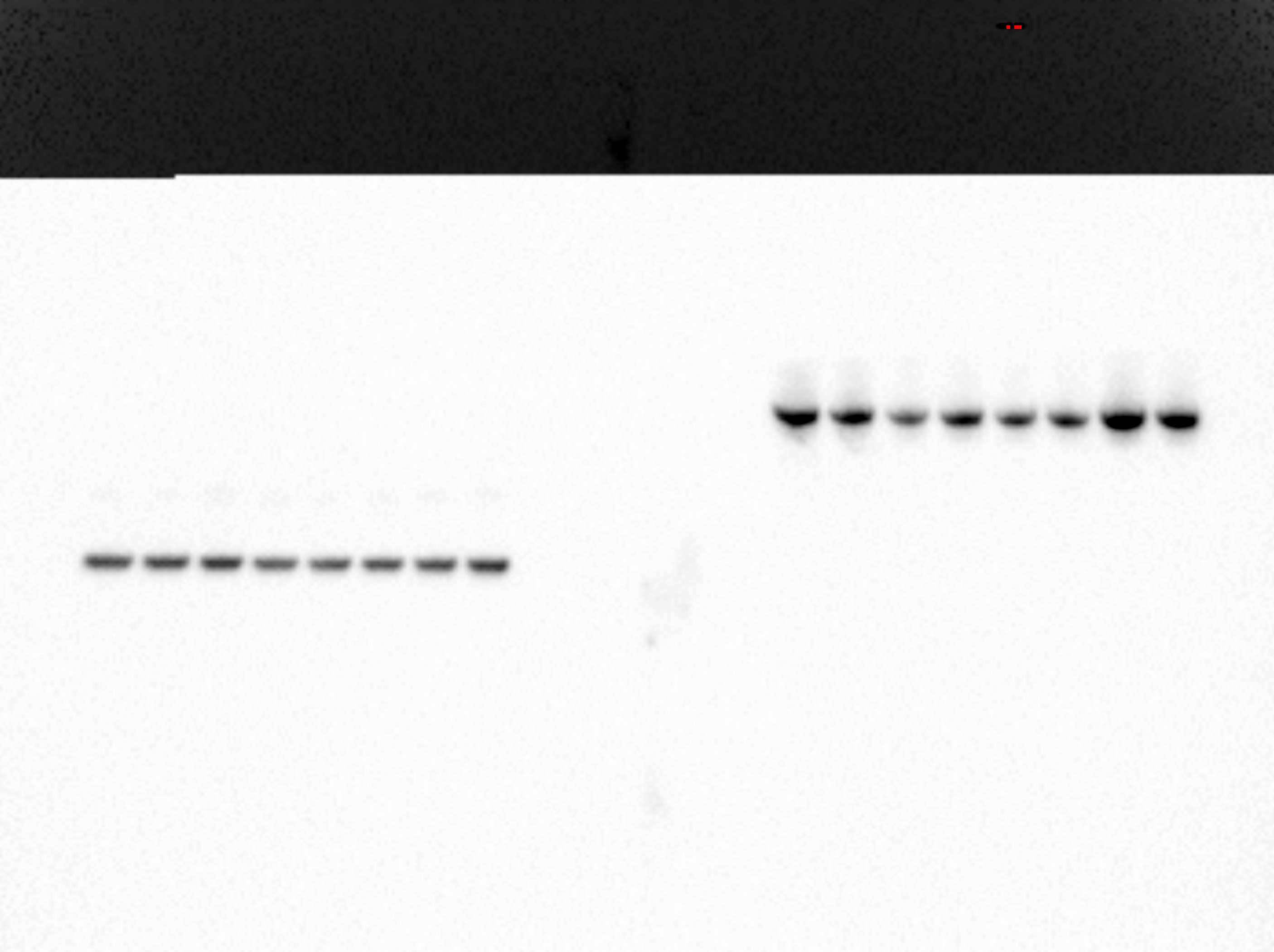

Supplement: Figure 6—source data 2. [file elife-104461-fig6-data2.zip › COX2 AND UCP1.tif]

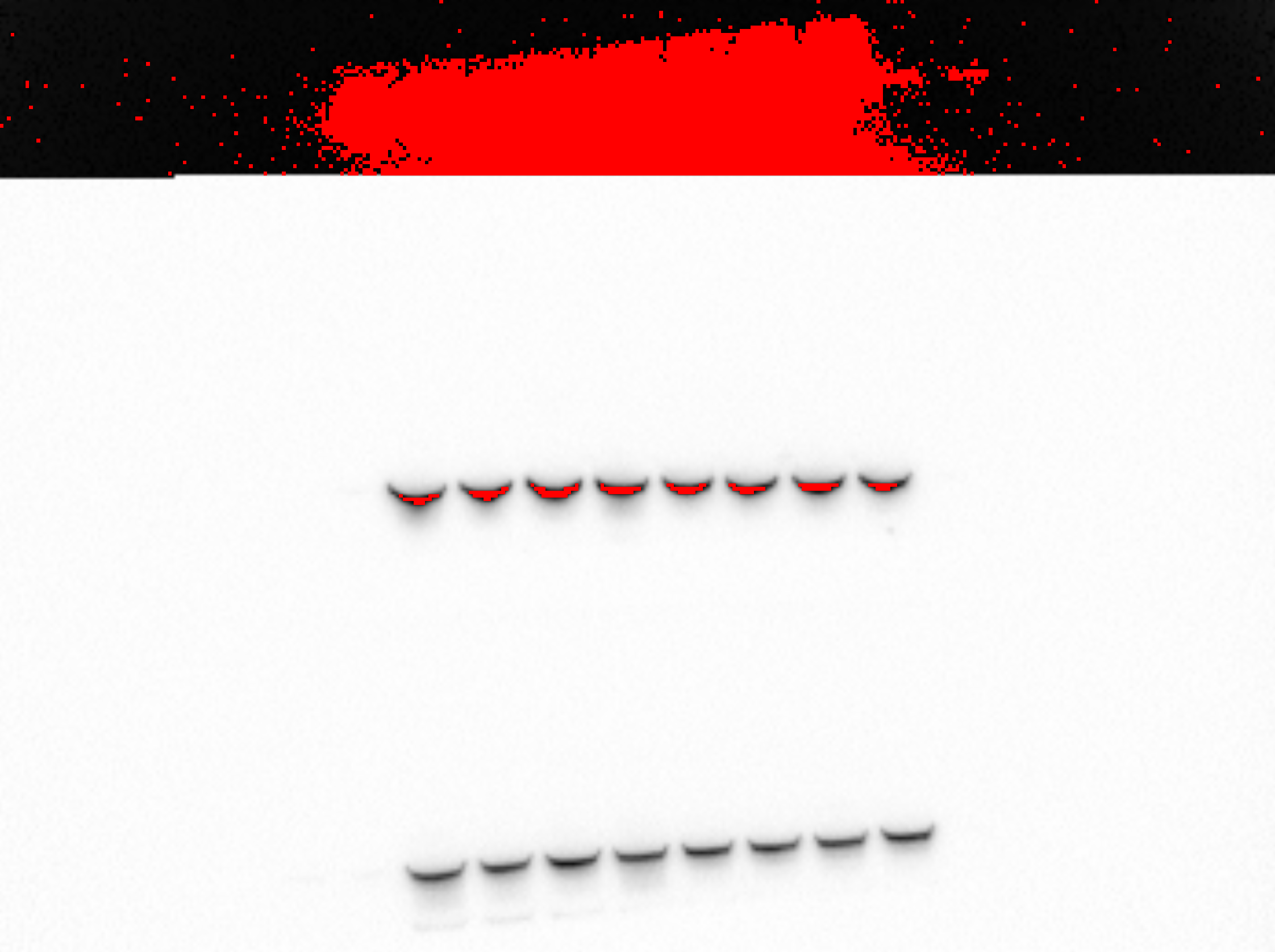

Supplement: Figure 6—source data 2. [file elife-104461-fig6-data2.zip › HSP60.tif]

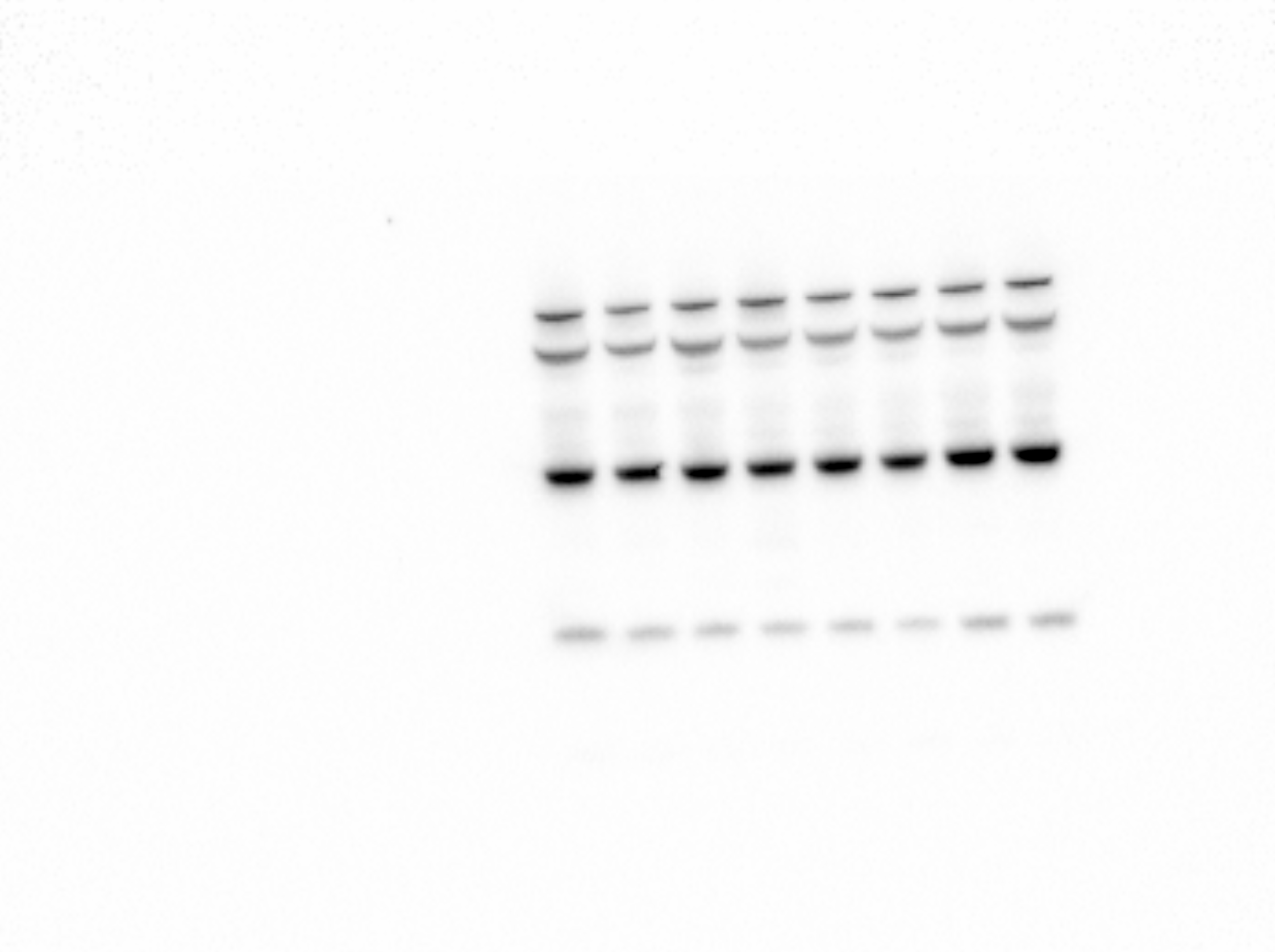

Supplement: Figure 6—source data 2. [file elife-104461-fig6-data2.zip › OXPHOS.tif]

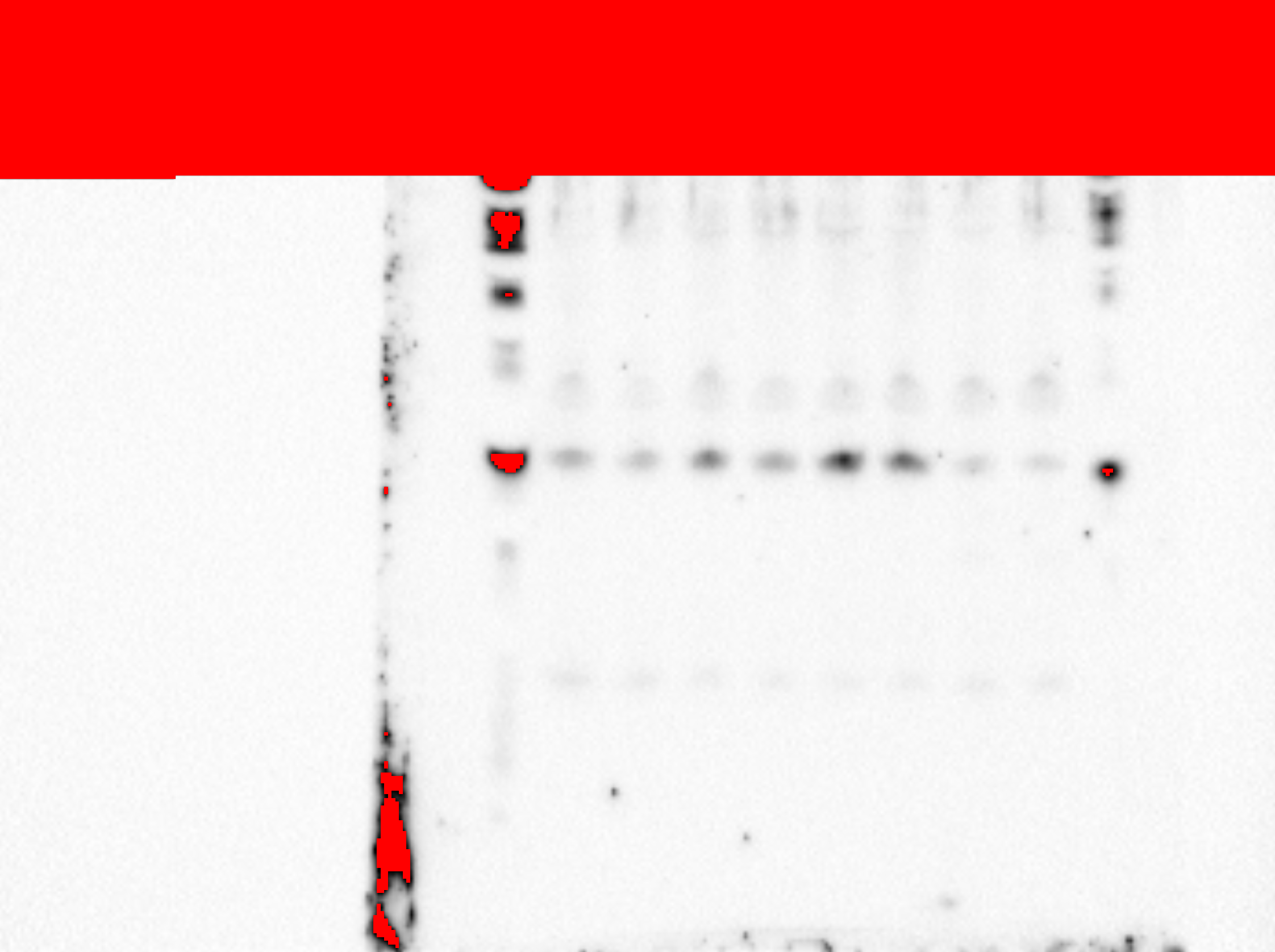

Supplement: Figure 6—source data 2. [file elife-104461-fig6-data2.zip › TFAM.tif]

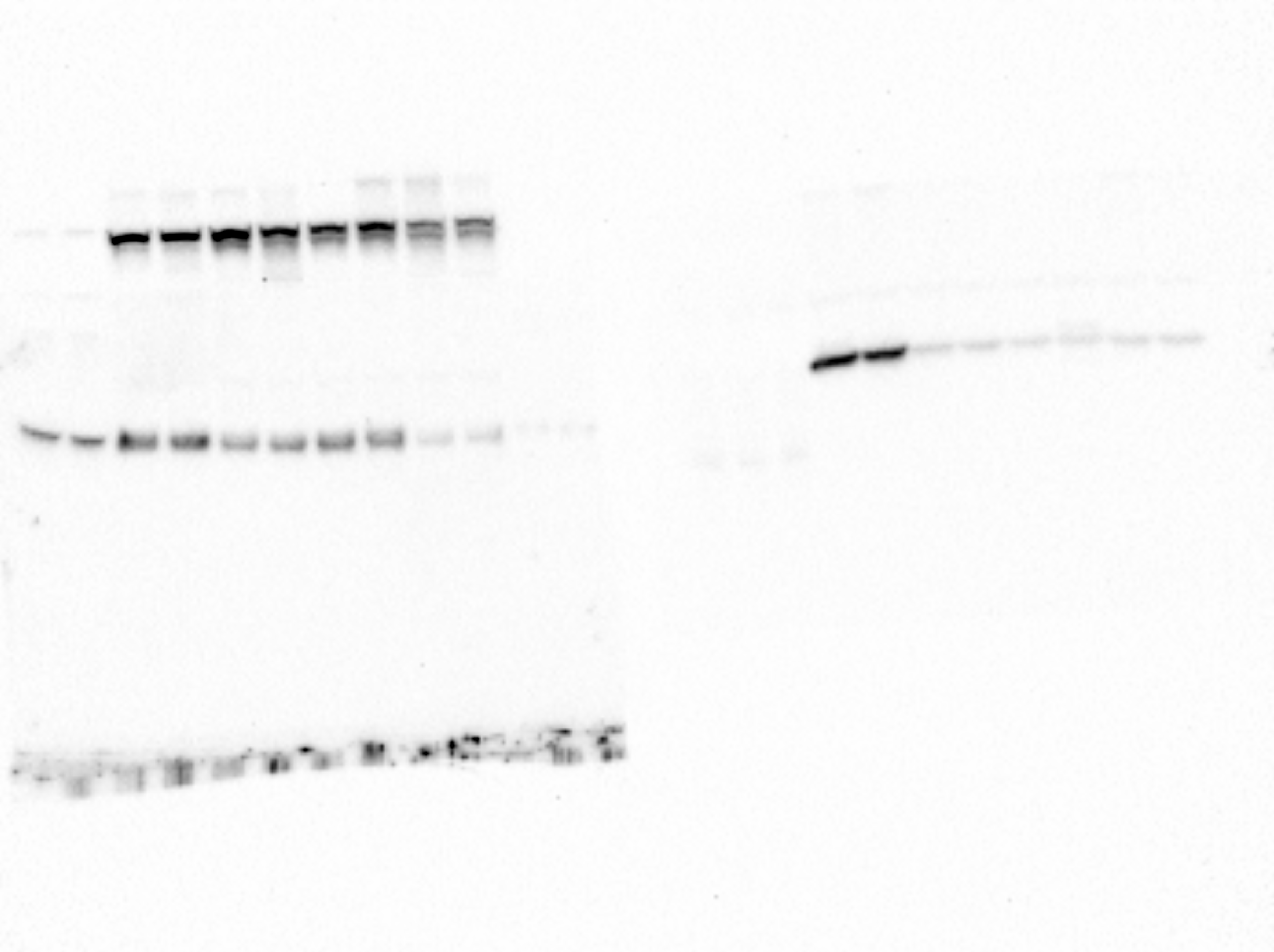

Supplement: Figure 7—source data 2. [file elife-104461-fig7-data2.zip › COX2.tif]

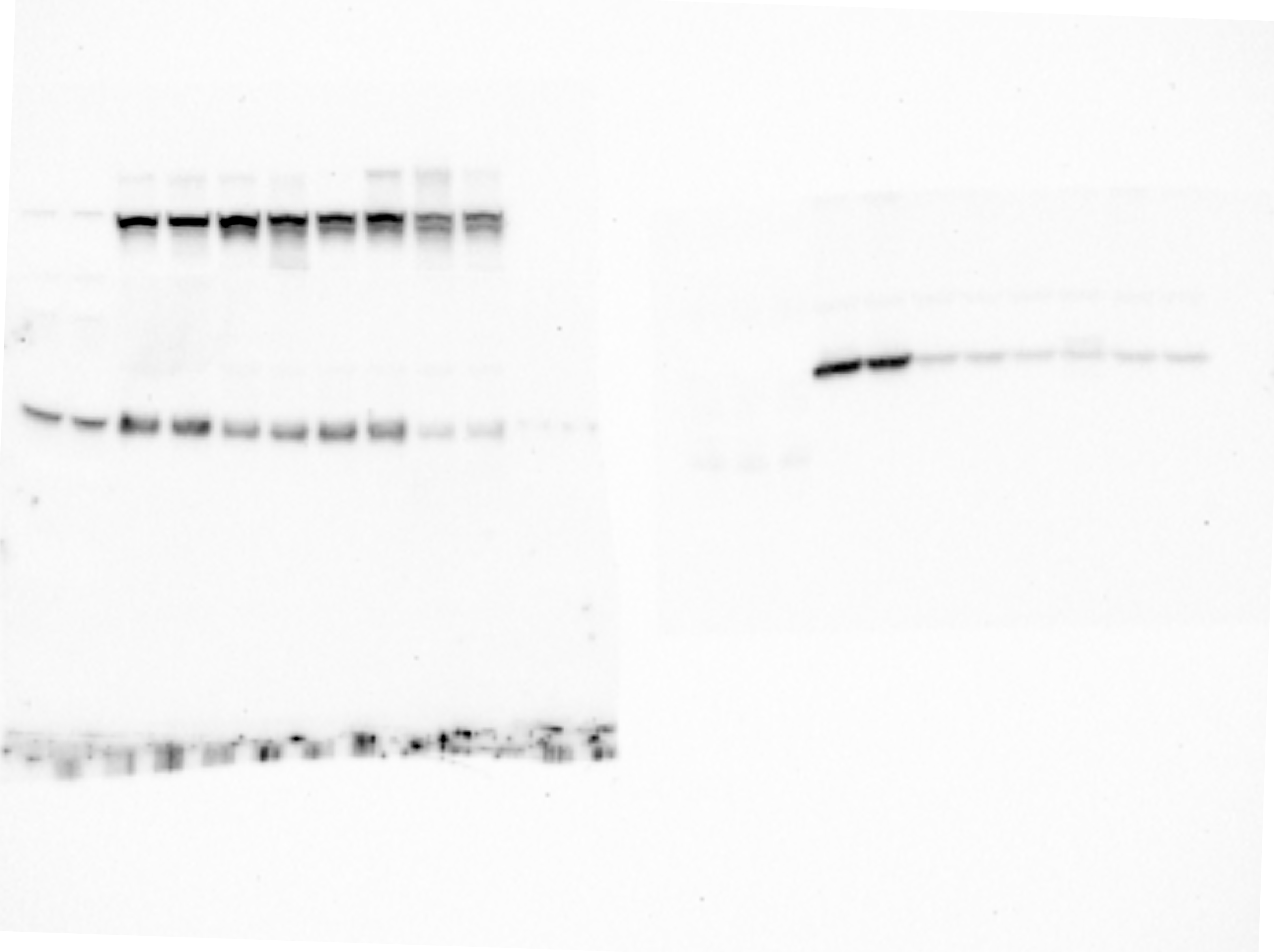

Supplement: Figure 7—source data 2. [file elife-104461-fig7-data2.zip › HSP60.tif]

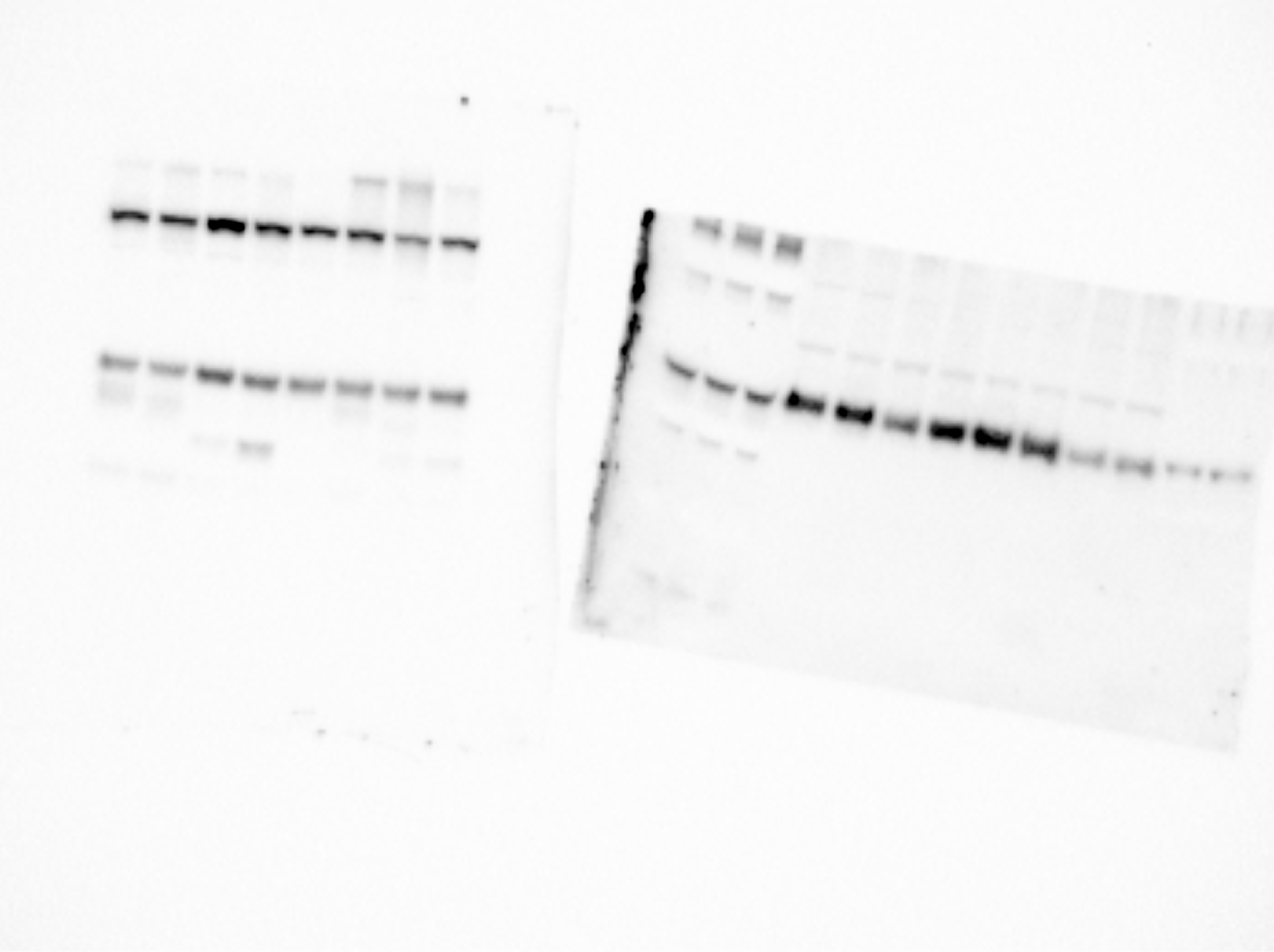

Supplement: Figure 7—source data 2. [file elife-104461-fig7-data2.zip › OXPHOS.tif]

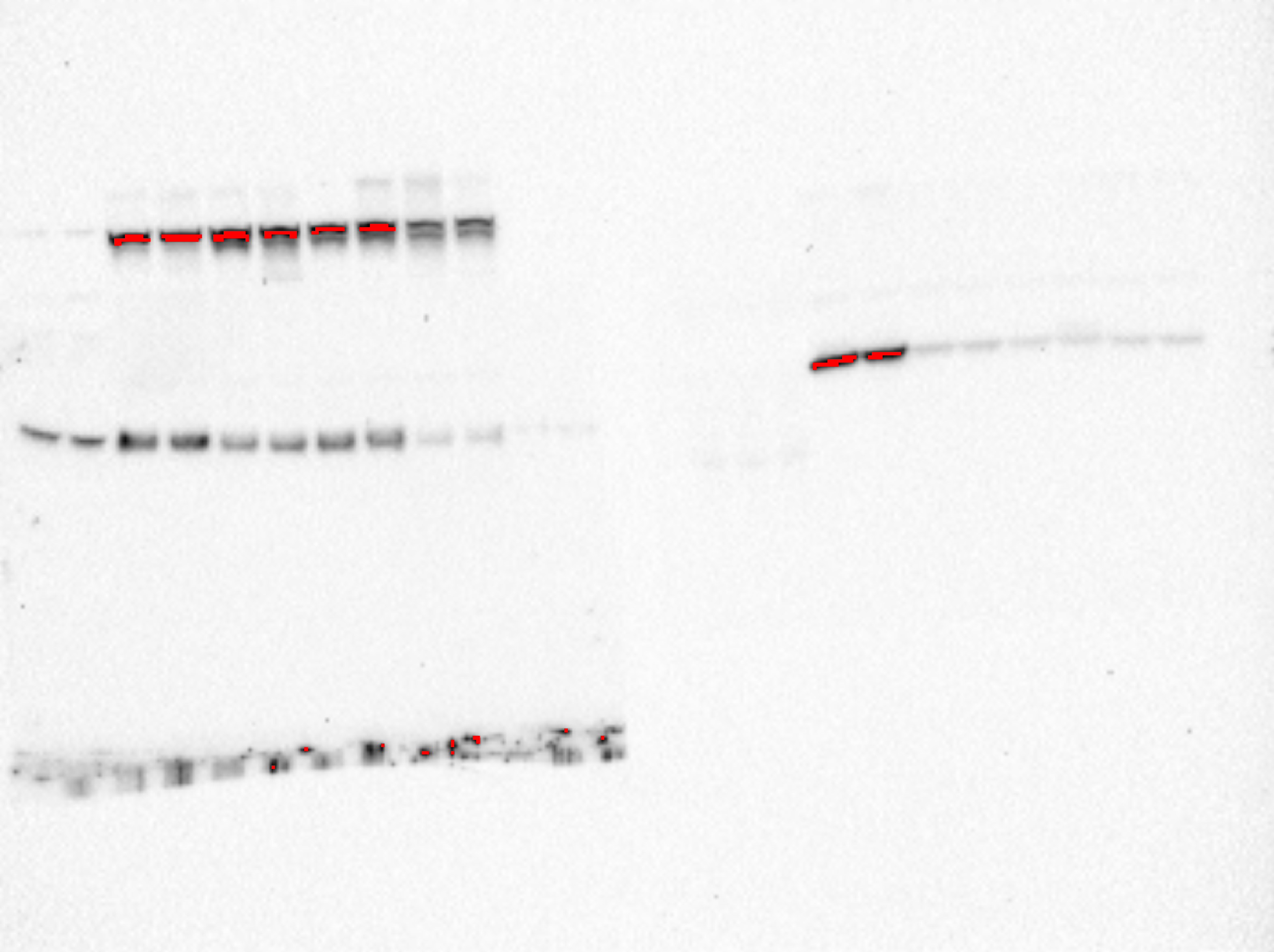

Supplement: Figure 7—source data 2. [file elife-104461-fig7-data2.zip › TFAM.tif]
