# Supplementary material for: Tissue-specific responses to TFAM and mtDNA copy number manipulation in prematurely ageing mice
Source: eLife. 2025 Jun 30;14:RP104461. doi: 10.7554/eLife.104461 (PMC12208663; doi:10.7554/eLife.104461)
Supplement: Figure 5—source data 5. [file elife-104461-fig5-data5.pdf]

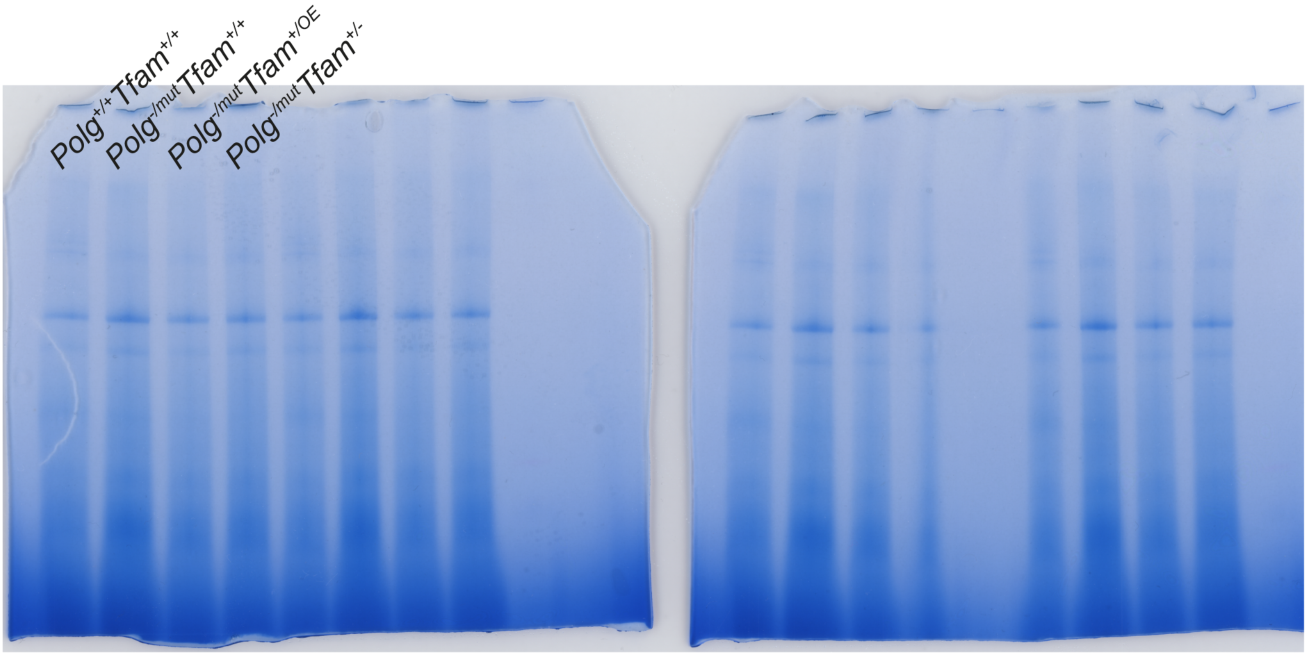

Commassie

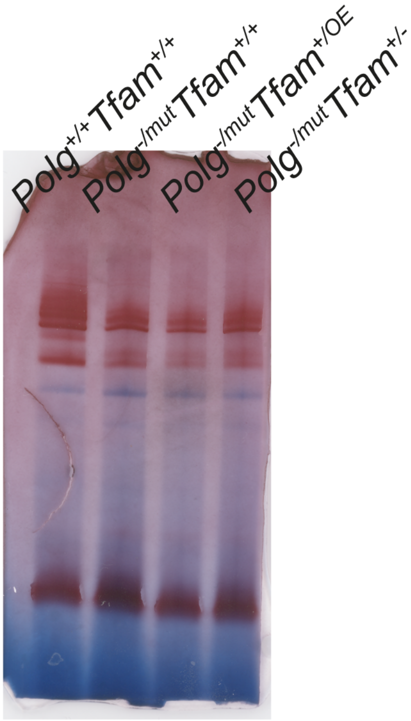

Complex I
in gel activity

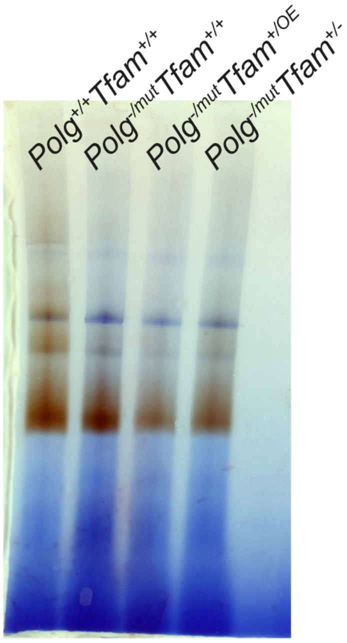

Complex IV
in gel activity

Figure 5 - Source data 5

BN-PAGE analysis of Figure 5C, labeled
