# Supplementary material for: Tissue-specific responses to TFAM and mtDNA copy number manipulation in prematurely ageing mice
Source: eLife. 2025 Jun 30;14:RP104461. doi: 10.7554/eLife.104461 (PMC12208663; doi:10.7554/eLife.104461)
Supplement: Figure 7—source data 3. [file elife-104461-fig7-data3.pdf]

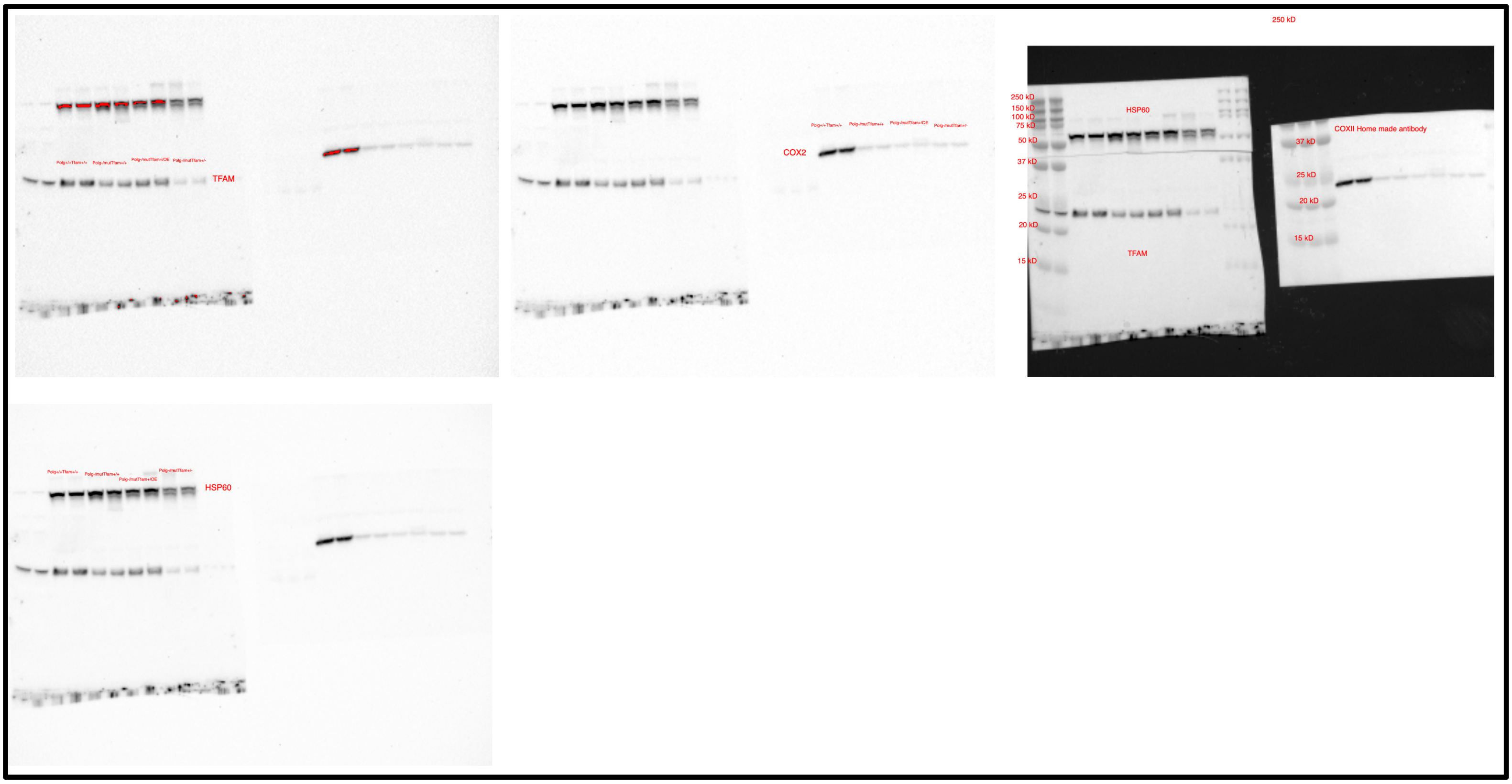

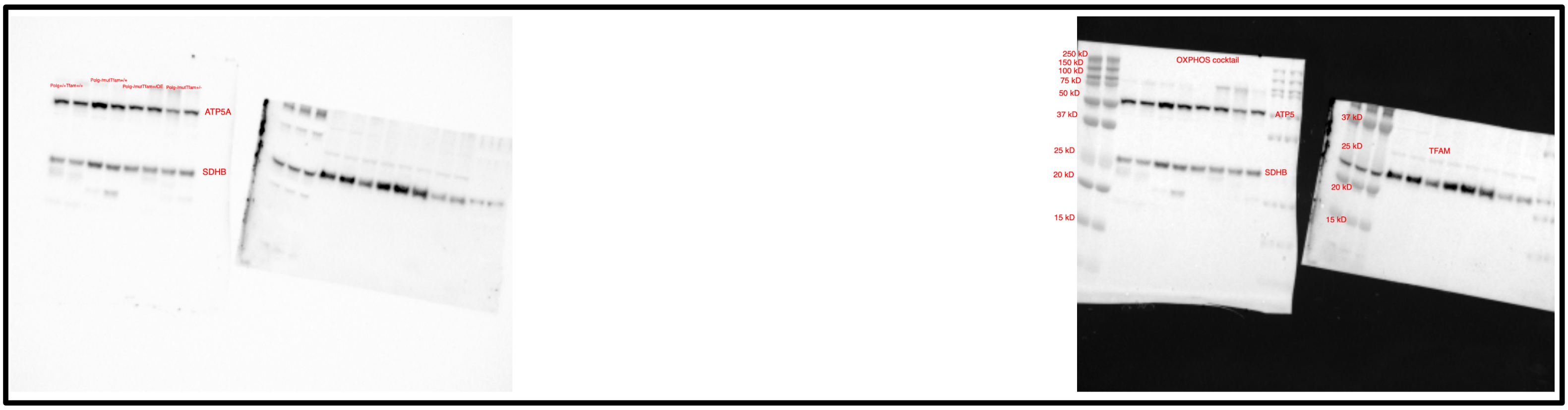

Figure 7 - Source data 3

Western blot analysis of Figure 7B, boxes indicating associated images, relevant bands in kilo Dalton (kD)
